# TrajPy: empowering feature engineering for trajectory analysis across domains

**DOI:** 10.1093/bioadv/vbae026

**Published:** 2024-02-23

**Authors:** Maurício Moreira-Soares, Eduardo Mossmann, Rui D M Travasso, José Rafael Bordin

**Affiliations:** Oslo Centre for Biostatistics and Epidemiology, University of Oslo, Oslo, 0373, Norway; Centre for Bioinformatics, University of Oslo, Oslo, 0373, Norway; School of Engineering and Computer Science, Victoria University of Wellington, Wellington, 6012, New Zealand; Department of Physics, Institute of Physics and Mathematics, Universidade Federal de Pelotas, Pelotas, 96160-000, Brazil; CFisUC, Department of Physics, University of Coimbra, Coimbra, 3004-516, Portugal; Department of Physics, Institute of Physics and Mathematics, Universidade Federal de Pelotas, Pelotas, 96160-000, Brazil

## Abstract

**Motivation:**

Trajectories, which are sequentially measured quantities that form a path, are an important presence in many different fields, from hadronic beams in physics to electrocardiograms in medicine. Trajectory analysis requires the quantification and classification of curves, either by using statistical descriptors or physics-based features. To date, no extensive and user-friendly package for trajectory analysis has been readily available, despite its importance and potential application across various domains.

**Results:**

We have developed TrajPy, a free, open-source Python package that serves as a complementary tool for empowering trajectory analysis. This package features a user-friendly graphical user interface and offers a set of physical descriptors that aid in characterizing these complex structures. TrajPy has already been successfully applied to studies of mitochondrial motility in neuroblastoma cell lines and the analysis of *in silico* models for cell migration, in combination with image analysis.

**Availability and implementation:**

The TrajPy package is developed in Python 3 and is released under the GNU GPL-3.0 license. It can easily be installed via PyPi, and the development source code is accessible at the repository: https://github.com/ocbe-uio/TrajPy/. The package release is also automatically archived with the DOI 10.5281/zenodo.3656044.

## 1 Introduction

Trajectories are present in several fields of science with varying definitions but are intuitively understood as a set of points sequentially ordered and interconnected forming a path. More rigorously, a trajectory is defined by a sequence ${\left(x_{n}\left(t_{n}\right)\right)}_{n\geq 0}$ of values x, measured at time $t_{n}$ with ordering index n that is ≥0 (*n* ≥ 0)

$${\left(x_{n}\left(t_{n}\right)\right)}_{n\geq 0}=x_{0}(t_{0}),\;x_{1}(t_{1}),x_{2}(t_{2}),\;\ldots .$$

This sequence may be obtained experimentally or be the result of a numerical calculation, may follow a closed mathematical form, a recursive definition, or obey a physical law or a biological mechanism (Levin 2021). The values of x are often spatial coordinates in the Euclidean space, but any quantity measured repeatedly can delineate a trajectory in an abstract space, such as blood pressure (Ji *et al.* 2020), sunburn incidence (Lergenmuller *et al.* 2022), or physical activity recorded over time (Perrier *et al.* 2022).

Considerable effort has been employed to characterize complex trajectories in biology at different scales, from the diffusion of proteins and nano particles at the subcellular level (Huet *et al.* 2006, Arcizet *et al.* 2008), through wandering ants and migratory birds (Wolf and Wehner 2000, Croxall *et al.* 2005) to the study of hand tremor trajectories in Parkinson’s disease (San-Segundo *et al.* 2020). Due to recent advances in microscopy, we can visualize the inner life of the cell, including the dynamics of mRNA, mitochondria, microtubules, actin filaments, etc. These rich trajectory data are challenging to summarize and to model in their raw format, demanding feature extraction and quantification. In biostatistics, trajectories arise naturally in clinical trials and observational longitudinal studies when more than two measurements are recorded for the same patient at different time points, such as in the studies on sunburns and physical activity previously mentioned. This type of data requires methods developed for the analysis of repeated measurements and can also benefit from feature engineering.

From the perspective of trajectory analysis in molecular dynamics (MD) simulations, among many available software, we highlight three packages: MDAnalysis (Michaud-Agrawal *et al.* 2011), PTraj/CPPTraj (Roe and Cheatham 2013), and freud (Ramasubramani *et al.* 2020). These packages are the state-of-the-art for MD analysis, but they require mastering either low-level programming or command-line interfaces, which may pose a challenge for the end-user. Moreover, they are not suitable for general applications purposes.

The main goal of well-established methods, such as trackpy (http://soft-matter.github.io/trackpy/), lies in image processing, and these codes are heavily oriented toward specific, field-dependent needs. They provide a limited number of quantitative descriptors, often with focus only on the net displacement or the mean squared displacement (MSD) for estimating the diffusion exponent (Allan *et al.* 2023). However, these measures lack sensitivity for the characterization of different kinds of trajectories in biophysics (Burnecki *et al.* 2015). Therefore, there is a demand for specialized packages that aim to improve and democratize feature engineering for general trajectory analysis.

We propose TrajPy as a framework for tackling these challenges, aiming at broad applications across domains. The package can be integrated at the tip of image analysis pipelines, used for postprocessing of *in silico* simulations or longitudinal clinical data.

Successful modern methods perform the computation of physical properties related to the kinematics and/or morphology of the curves, to build a multidimensional space of attributes. This step allows for the unveiling of hidden information about the trajectories by applying multivariate machine-learning methods. For instance, in Wagner *et al.* (2017), the authors propose a set of features to quantify single cell dynamics and draw conclusions regarding the classification of cell movement. They provide a random forest classifier TraJClassifier as plugin for the image analysis software Fiji (Schindelin *et al.* 2012). The attributes selected in this work are known in physics as good predictors for classifying movements in different types (sub-diffusion, normal diffusion, super-diffusion, and anomalous diffusion), but beyond these classifications they are also helpful for identifying key differences between trajectories, even under the same diffusion regime.

We expanded the set of features proposed by Wagner *et al.* with Fourier analysis and improved the estimation of the diffusion coefficient by implementing the Green–Kubo method. TrajPy currently offers 17 features that can be computed for any generic trajectory. It is important to note that TrajPy is not intended to replace specialized software, but it was developed as a building block that can work in synergy with other field-specific methods. In addition, it comes with a user-friendly graphical user interface (GUI) that requires no programming skills, making it accessible for experts in different fields to empower data analyses.

## 2 Methods

**Figure 1.**
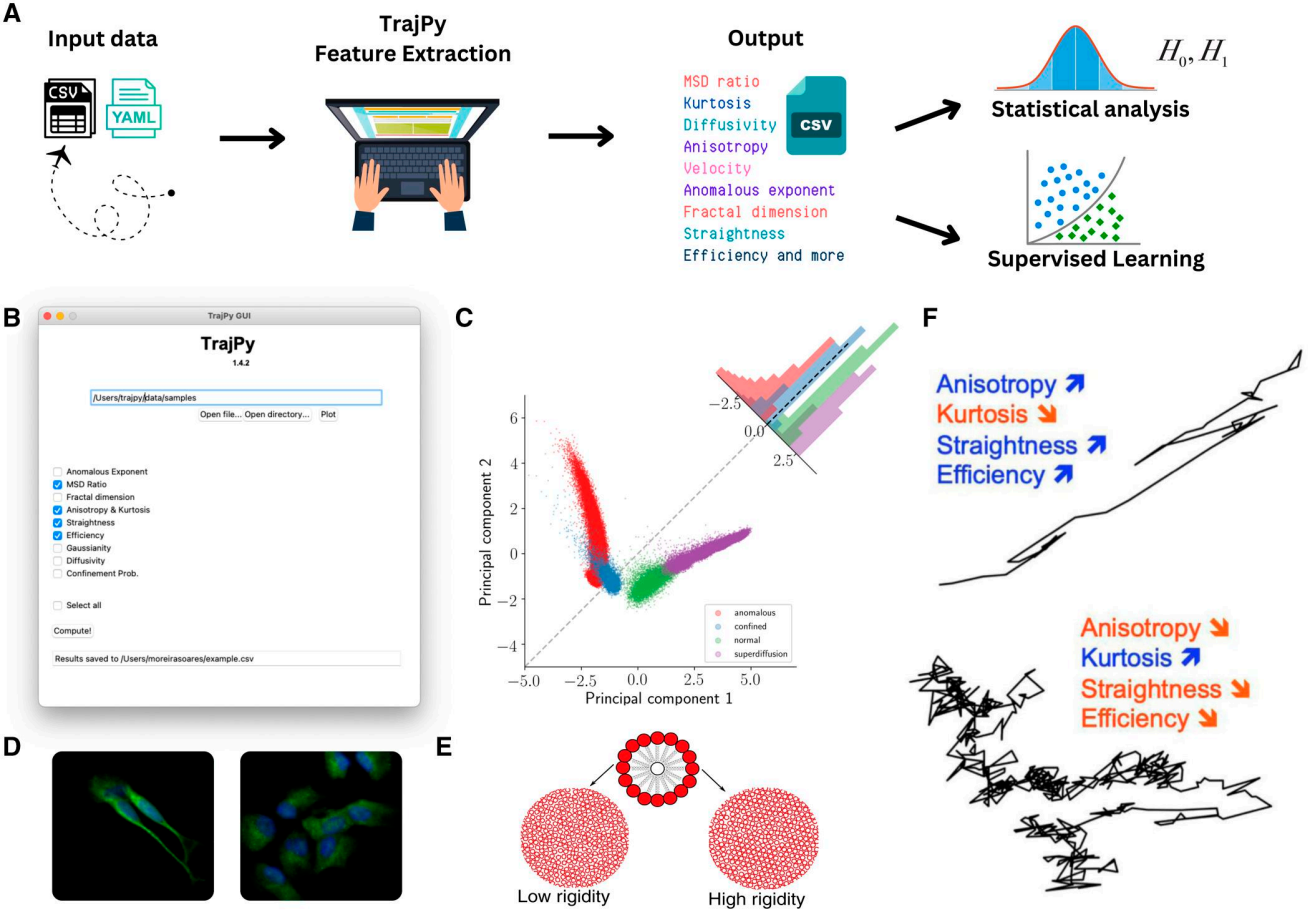
Applications and functionalities. (A) The typical workflow includes reading trajectories from CSV or YAML files, perform feature extraction with TrajPy, save the data as CSV, and either conduct classical statistical analysis (e.g. hypothesis testing) or supervised learning if curated labels are available. In (B), we present TrajPy’s GUI. In (C), PCA projection of trajectory data showing four well-separated clusters, colored by true labels, validating the discriminative power of TrajPy’s feature set. (D) Neuroblastoma-derived cell line utilized for studying mitochondrial motility. (E) A system of bead-spring polymeric entities employed as an *in silico* model for biological cells. For both (D) and (E), dynamics were quantified using TrajPy’s features, with the input being the trajectories extracted from experimental videos and simulations, respectively. In (F), the change in a set of four features from TrajPy between two distinguished trajectories is depicted. (C) and (F), (D), and (E) were adapted from Simões *et al.* (2021), Soares (2020), and Mossman (2022), respectively.

TrajPy is an open-source python package in continuous development on GitHub that welcomes external contributions. We employ continuous integration/continuous deployment with automated unit tests to assure code quality and reliability. All releases are published automatically to the PyPi repository, offering a simple method for installation and dependency management. The package development is driven by the aim of long-term maintainability and, as such, the number of external dependencies is kept at bare minimum. The core engine of the package requires only the standard packages for scientific computing *scipy* and *numpy*. In addition, to run the GUI the packages *ttkthemes* and *Pillow* are needed. Furthermore, *PyYAML* provides support for parsing MD simulation data from LAMMPS (Plimpton *et al.* 2021). We provide online documentation with *readthedocs*. Figure 1A illustrates the typical sequence of steps in the workflow when using TrajPy.

TrajPy consists of three main units of code, as described below. The heart of the package lies in *trajpy.py* and contains the class *Trajectory*, which can be initialized either as a dummy object for calling its functions, or by loading a trajectory array, or a CSV trajectory file. This primary code allows the user to compute the various physical and statistical attributes, such as the MSD, diffusion coefficient and velocity of any given trajectory (see Supplementary Table S1 for the extensive list of features). The second unit, *traj_generator.py*, consists of a collection of methods implemented to simulate different diffusion modes: confined, normal, anomalous, and direct motion. Lastly, but not least important, the *gui.py* contains the code for running the GUI, which provides a friendly interface that requires no knowledge of programming from the user (see Fig. 1B).

We propose two independent and complementary workflows for data analysis with TrajPy. The first approach encompasses the development of a classification model for the diffusion modes aforementioned. We generate synthetic trajectories by employing four independent simulation engines that generate trajectories on each one of the four labels (sub-diffusion, normal diffusion, super-diffusion, and anomalous diffusion). The space of parameters for these simulations can be explored to obtain different trajectories that obey the same diffusion regime. Then, we apply feature engineering to quantify these trajectories with the proposed features in TrajPy. The data generated with the features and the labels are used to train a classifier that can be used later for classifying unseen data generated from simulations or experiments. We provide a dataset of synthetic data that can be used to train new models (Moreira-Soares 2020). Figure 1C depicts the principal components for the synthetic data and the diffusion modes clusters (Soares 2020).

The second workflow focuses on the statistical analysis of raw, unlabeled experimental trajectory data. Using the same feature engineering techniques as in the first workflow, we extract identical attributes from the experimental data, those which were previously used to train our classifier. While these experimental trajectories are not subjected to classification, the derived features remain invaluable for quantifying and analyzing diverse biological systems in various research domains. Through statistical inference, these features facilitate the extraction of new insights into the underlying dynamics of the systems under study. For example, the features enable hypothesis testing to discern significant differences between control groups and those subjected to specific treatments.

In addition, new classifiers can be trained based on labels that may be of interest in other fields, depending on domain knowledge. For example, water quality conditions affect fish trajectories, so classifying these trajectories between “Normal water quality” and “Polluted/abnormal water quality” is more relevant than diffusion modes classification in this context (Cheng *et al.* 2019).

## 3 Validation and results

The package was applied to study neuronal mitochondrial trafficking in neuroblastoma cell lines (Simões *et al.* 2021). In this study, the researchers exposed the cells to mitochondrial toxins and recorded the mitochondrial trajectories using TIRF microscopy (see Fig. 1D). By characterizing the dynamic trajectories, they analyzed how mitochondrial motility was affected. The application of TrajPy’s feature engineering facilitated a deeper understanding of the underlying biological process. The findings revealed a novel quantitative approach describing how mitochondria behave in both healthy and diseased neuronal cells, demonstrating the valuable potential of TrajPy for applications in the study of subcellular dynamics.

In another study, TrajPy was employed to quantify and analyze the migration behavior of self-propelled droplets in dense fibrous media modelled *in silico* (Moreira-Soares *et al.* 2020). By using TrajPy’s feature engineering capabilities, the velocity of the cells and morphology of the cell’s trajectory were measured, as a function of fiber density and adhesiveness between the cell and the matrix fibers. TrajPy enabled the comparison of simulation results with *in vitro* migration assay data of fibrosarcoma cells in fibrous matrices, demonstrating good agreement between the two methodologies. This study shed light on the critical role of adhesiveness in cell migration within crowded environments.

Moreover, TrajPy has been used to explore the behavior of a simplified drop-like model representing biological cells as it undergoes the jamming transitions (Mossmann 2022), the physical process by which viscosity increases with increasing particle density (Mongera *et al.* 2018). In Fig. 1E, we can see the cell model with deformable boundaries and the system under two conditions with low and high rigidity. The jamming transitions have recently been recognized as key in various biological processes, including cell migration, embryo development, tissue homeostasis, and disease progression (Sadati *et al.* 2014, Oswald *et al.* 2017, Lenne and Trivedi 2022, Gottheil *et al.* 2023). By utilizing TrajPy, the behavior of the drop-like model was quantified and analyzed as pressure was increased, leading to the change in fluid viscosity. In addition, the cells trajectories were classified into the four diffusion modes using a classifier built with TrajPy’s synthetic data. Through the application of TrajPy, the project gained valuable insights into how cell populations rapidly and significantly change their material properties during jamming transition, revealing the physiological relevance of these transition and permitting to explore potential regulatory mechanisms.

Figure 1F gives an insight into how a set of four features implemented in TrajPy change between a trajectory with high persistency time (upper) and another with higher stochasticity (lower). More examples are provided in the Supplementary Material, in the package’s documentation, and in the code repository.

## Supplementary Material

vbae026_Supplementary_Data

## Data and code availabiity

The data underlying this article are available in the article and in its online supplementary material. The code for TrajPy is available in Zenodo, at https://doi.org/10.5281/zenodo.8262793.

## References

[vbae026-B1] Allan DB , Caswell T, Keim NC et al soft-matter/trackpy: V0. 2023. 10.5281/zenodo.1213240 (23 October 2023, date last accessed).

[vbae026-B2] Arcizet D , Meier B, Sackmann E et al Temporal analysis of active and passive transport in living cells. Phys Rev Lett 2008;101:248103. 10.1103/PhysRevLett.101.248103 19113674

[vbae026-B3] Burnecki K , Kepten E, Garini Y et al Estimating the anomalous diffusion exponent for single particle tracking data with measurement errors-an alternative approach. Sci Rep 2015;5:11306. 10.1038/srep11306 26065707 PMC4463942

[vbae026-B4] Cheng S , Zhao K, Zhang D. Abnormal water quality monitoring based on visual sensing of three-dimensional motion behavior of fish. Symmetry 2019;11:1179. 10.3390/sym11091179

[vbae026-B5] Croxall JP , Silk JRD, Phillips RA et al Global circumnavigations: tracking year-round ranges of nonbreeding albatrosses. Science 2005;307:249–50. 10.1126/science.1106042 15653503

[vbae026-B6] Gottheil P , Lippoldt J, Grosser S et al State of cell unjamming correlates with distant metastasis in cancer patients. Phys Rev X 2023;13:031003. 10.1103/PhysRevX.13.031003

[vbae026-B7] Huet S , Karatekin E, Tran VS et al Analysis of transient behavior in complex trajectories: application to secretory vesicle dynamics. Biophys J 2006;91:3542–59. 10.1529/biophysj.105.080622 16891360 PMC1614485

[vbae026-B8] Ji H , Kim A, Ebinger JE et al Sex differences in blood pressure trajectories over the life course. JAMA Cardiol 2020;5:19–26. 10.1001/jamacardio.2019.5306 31940010 PMC6990675

[vbae026-B9] Lenne P-F , Trivedi V. Sculpting tissues by phase transitions. Nat Commun 2022;13:664. 10.1038/s41467-022-28151-9 35115507 PMC8814027

[vbae026-B10] Lergenmuller S , Rueegg CS, Perrier F et al Lifetime sunburn trajectories and associated risks of cutaneous melanoma and squamous cell carcinoma among a cohort of Norwegian women. JAMA Dermatol 2022;158:1367–77. 10.1001/jamadermatol.2022.4053 36197657 PMC9535508

[vbae026-B11] Levin O. Discrete Mathematics: An Open Introduction. 3rd edn. Greeley, Colorado, United States, 2021. https://discrete.openmathbooks.org (12 October 2023, date last accessed).

[vbae026-B12] Michaud-Agrawal N , Denning EJ, Woolf TB et al MDAnalysis: a toolkit for the analysis of molecular dynamics simulations. J Comput Chem 2011;32:2319–27. 10.1002/jcc.21787 21500218 PMC3144279

[vbae026-B13] Mongera A , Rowghanian P, Gustafson HJ et al A fluid-to-solid jamming transition underlies vertebrate body axis elongation. Nature 2018;561:401–5. 10.1038/s41586-018-0479-2 30185907 PMC6148385

[vbae026-B14] Moreira-Soares M. Training dataset for trajectory classification. 2020. 10.5281/zenodo.3627650 (23 October 2023, date last accessed).

[vbae026-B15] Moreira-Soares M , Cunha SP, Bordin JR et al Adhesion modulates cell morphology and migration within dense fibrous networks. J Phys Condens Matter 2020;32:314001. 10.1088/1361-648X/ab7c17 32378515

[vbae026-B16] Mossmann E. A Physics Based Feature Engineering Framework for Trajectory Analysis. Pelotas, Rio Grande do Sul, Brazil: Universidade Federal de Pelotas, 2022. https://sucupira.capes.gov.br/sucupira/public/consultas/coleta/trabalhoConclusao/viewTrabalhoConclusao.jsf?popup=true&id_trabalho=11667233# (27 October 2023, date last accessed).

[vbae026-B17] Oswald L , Grosser S, Smith DM et al Jamming transitions in cancer. J Phys D Appl Phys 2017;50:483001. 10.1088/1361-6463/aa8e83 29628530 PMC5884432

[vbae026-B18] Perrier F , Ghiasvand R, Lergenmuller S et al Life-course trajectories of physical activity and melanoma risk in a large cohort of Norwegian women. Clin Epidemiol 2022;14:1571–84. 10.2147/CLEP.S382454 36578536 PMC9791937

[vbae026-B19] Plimpton S , Kohlmeyer A, Thompson A et al LAMMPS stable release 29 September 2021*.* 2021. 10.5281/zenodo.6386596 (23 October 2023, date last accessed).

[vbae026-B20] Ramasubramani V , Dice BD, Harper ES et al freud: a software suite for high throughput analysis of particle simulation data. Comput Phys Commun 2020;254:107275. 10.1016/j.cpc.2020.107275

[vbae026-B21] Roe DR , Cheatham TE. PTRAJ and CPPTRAJ: software for processing and analysis of molecular dynamics trajectory data. J Chem Theory Comput 2013;9:3084–95. 10.1021/ct400341p 26583988

[vbae026-B22] Sadati M , Nourhani A, Fredberg JJ et al Glass‐like dynamics in the cell and in cellular collectives. Wiley Interdiscip Rev Syst Biol Med 2014;6:137–49. 10.1002/wsbm.1258 24431332 PMC4000035

[vbae026-B23] San-Segundo R , Zhang A, Cebulla A et al Parkinson’s disease tremor detection in the wild using wearable accelerometers. Sensors 2020;20:5817. 10.3390/s20205817 33066691 PMC7602495

[vbae026-B24] Schindelin J , Arganda-Carreras I, Frise E et al Fiji: an open-source platform for biological-image analysis. Nat Methods 2012;9:676–82. 10.1038/nmeth.2019 22743772 PMC3855844

[vbae026-B25] Simões RF , Pino R, Moreira‐Soares M et al Quantitative analysis of neuronal mitochondrial movement reveals patterns resulting from neurotoxicity of rotenone and 6‐hydroxydopamine. FASEB J 2021;35:e22024. 10.1096/fj.202100899R 34751984

[vbae026-B26] Soares MM. Multi-phase-field models for biological systems: from cells to vessels. Unpublished Ph.D. Thesis, University of Coimbra, 2020. https://hdl.handle.net/10316/96232 (23 October 2023, date last accessed).

[vbae026-B27] Wagner T , Kroll A, Haramagatti CR et al Classification and segmentation of nanoparticle diffusion trajectories in cellular micro environments. PLoS One 2017;12:e0170165. 28107406 10.1371/journal.pone.0170165 PMC5249096

[vbae026-B28] Wolf H , Wehner R. Pinpointing food sources: olfactory and anemotactic orientation in desert ants, Cataglyphis fortis. J Exp Biol 2000;203:857–68. 10.1242/jeb.203.5.857 10667968

