# Supplementary material for: TrajPy: empowering feature engineering for trajectory analysis across domains
Source: Bioinform Adv. 2024 Feb 23;4(1):vbae026. doi: 10.1093/bioadv/vbae026 (PMC11032726; doi:10.1093/bioadv/vbae026)
Supplement: vbae026_Supplementary_Data [file vbae026_supplementary_data.pdf]

Supplementary information of: *"TrajPy: empowering feature engineering for trajectory analysis across domains"*

Maurício Moreira-Soares[1,2,*]
m.m.soares@medisin.uio.no

Eduardo Henrique Mossmann[3,4]
eduardo.mossmann@ecs.vuw.ac.nz

Rui D. M. Travasso[5]
ruit@uc.pt

José Rafael Bordin[4]
jrbordin@ufpel.edu.br

[1]Oslo Centre for Biostatistics and Epidemiology, University of Oslo, Oslo, Norway.
[2]Centre for Bioinformatics, University of Oslo, Oslo, Norway.
[3]School of Engineering and Computer Science, Victoria University of Wellington, Wellington, New Zealand.
[4]Department of Physics, Institute of Physics and Mathematics, Universidade Federal de Pelotas, Pelotas, Brazil.
[5]CFisUC, Department of Physics, University of Coimbra, Coimbra, Portugal.

January 2024

Contents

1 **Installation** 3

2 **Basic usage** 3
2.1 Graphical User Interface (GUI) 3
2.2 File formats 3
2.2.1 Comma separated values (CSV) 3
2.2.2 LAMMPS YAML dump format 4

3 **Physical concepts of interest** 4

4 **Trajectory quantification** 10

5 **Trajectory simulations** 24

1 Installation

The package is hosted on PyPi. To install it, use the following command line:

```
pip3 install trajpy
```

To test the development version, clone the repository into your local directory from your terminal using this command:

```
git clone https://github.com/ocbe-uio/trajpy
```

After that, run the setup.py file to install it:

```
python setup.py --install
```

2 Basic usage

2.1 Graphical User Interface (GUI)

To access the GUI, open a terminal and run the following line:

```
python3 -m trajpy.gui
```

If this command executes successfully, a new window will open, as shown in Figure 1B of the main text.

Here are some basic steps to start using the GUI:

1. Click on `Open file...` to process a single file, or use `Open directory...` to process multiple files in the same directory.
2. Select the features you want to compute by checking the appropriate boxes.
3. Click `Compute` to initiate processing.
4. Specify the directory and filename to save the results.

Processing is complete when the following message appears in the message box located at the bottom of the GUI:

```
Results saved to /path/to/results/output.csv
```

2.2 File formats

2.2.1 Comma separated values (CSV)

Currently, trajpy supports CSV files that are organized with four columns representing time t and three spatial coordinates x, y, and z. The first row of the CSV file must contain the column names, serving as a header. Each subsequent row should correspond to a data entry where the values align with the respective columns named in the header. Below is an example illustrating a valid CSV file structure:

t	x	y	z
1.00	10.00	50.00	50.00
2.00	11.00	50.00	50.00
3.00	11.00	50.00	50.00
4.00	12.00	50.00	50.00
5.00	12.00	50.00	50.00
6.00	13.00	50.00	50.00

See the sample file provided in the repository for an example.

2.2.2 LAMMPS YAML dump format

LAMMPS YAML dump files are structured as follows:


```
---
time: 0.0
natoms: 100
keywords: [id, type, x, y, z, vx, vy, vz, fx, fy, fz]
data:
- [1, 1, 0.0, 0.0, 0.0, 0.0, 0.0, 0.0, -nan, -nan, -nan]
- [2, 1, 0.0, 0.0, 0.0, 0.0, 0.0, 0.0, -nan, -nan, -nan]
- [3, 1, 0.0, 0.0, 0.0, 0.0, 0.0, 0.0, -nan, -nan, -nan]
...
```


We support parsing of these data files with the function `parse_lammps_dump_yaml()` which is available in auxiliary_functions.py.

3 Physical concepts of interest

In order to discuss the idea of diffusion and show where it comes from, we will use Fick's laws of diffusion, first proposed by [20]. To do that, we begin by providing the definition of two main concepts, the flux and concentration of matter in a system.

Simply put, the flux $\vec{J}$ of matter is defined as the amount of matter that crosses an area per unit of time, whereas the concentration C indicates the quantity of matter[1] located at a certain region of the system of interest [29].

We now make two basic assumptions about the behavior of the substance present in the system. First, we assume that the substance will avoid any location where C is considered large, since the more particles there are in a specific set of coordinates, the more difficult it is for the substance to pass through that area. Second, we assume that the conservation of matter holds true at all times [15]. This means that two measurements of the total mass at different times must yield the same result.

By using our first assumption, we see that the flux of matter goes in the opposite direction of the gradient of concentration, since the gradient points to

[1] In the present work, we will use interchangeably the words "matter" and "substance".

the direction where C grows larger. In order to express this proportionality between $\vec{J}$ and C as an equation, we must use a constant.

We will call such a constant the "diffusion" of matter through the system. By doing that, we arrive at what is known as Fick's first law. This law is defined as

$$\vec{J} = -D\nabla C(r,t), \tag{1}$$

where D is the diffusion coefficient that tells us that diffusion is the movement of matter from a region of high concentration C to a region of low concentration. By dimensional analysis, we notice that the diffusion D is written in units of $\mathrm{m}^2 \cdot \mathrm{s}^{-1}$. Also, it is clear that diffusion tells us how fast the substance can sweep out a unit of area.

The negative sign in Equation (1) indicates that the flux of the substance happens in the direction of the lowest concentration. In other words, the substance tends to move along the path of least resistance where, in this context, the resistance is provided by the collisions between the substance's and environment's particles.

Given that the particles are not bounded to stay fixed, it is easy to see that in a small region, the flux of particles going into the region from the left might be different from the flux going outside from the right. This means the concentration C changes according to the location and time, which means C is a function of r and t, $C(r,t)$. We may express the amount of substance present in this region per unit of time as the difference between the inward and outward fluxes $\nabla \cdot \vec{J}(r,t)$, combine it with the concept of concentration and rearrange the difference to obtain:

$$\frac{\partial C(r,t)}{\partial t} + \nabla \cdot \vec{J}(r,t) = 0, \tag{2}$$

which is the equations that ensures the conservation of mass.

Now we can substitute $\vec{J}$ in Equation (2) by substituting Equation (1) in Equation (2) to obtain:

$$\frac{\partial C(r,t)}{\partial t} - D\nabla^2 C(r,t) = 0. \tag{3}$$

Equation (3) is known as Fick's second law, also called the Diffusion Equation. This is the equation that governs every system where our two basic assumptions hold true. Given that the Laplacian of the concentration C, $\nabla^2 C(r,t)$, tells us that the value of C located at r is the average value of the concentration in the surrounding region, it is simple to see that Equation (3) states that C changes over time according to the nearby concentration. The higher the concentration in the vicinity of the point r, the faster C changes in r.

At this point, it is important to point out that, in the present work, we consider the applications of Fick's laws to the context of Self Diffusion, where the substance we discussed so far is composed of one kind of particle. [22]. To see an application of Fick's laws in the context of Interdiffusion, where the substance is composed of two or more kinds of particles, see [27].

Let us solve Equation (3) to obtain a relation for the concentration C. To do that, we apply the Dirac delta function [8] as a boundary condition:

$$C(r,0) = M\delta(r). \tag{4}$$

In Equation (4), we will set $M = 1$ for simplicity. Notice that, by computing the integral

$$\int_{-\infty}^{\infty} C(r,0)dr = \int_{-\infty}^{\infty} \delta(r)dr = 1, \tag{5}$$

we assure the conservation of mass[2].

We may use the Fourier Transforms method following the work of [29] in order to solve Equation (3). The solution is given by

$$C(r,t) = \frac{1}{(4\pi Dt)^{n/2}} \exp\left(-r^2/4Dt\right), \tag{6}$$

where n is the number of dimensions of the system.

Since Equation (6) is a Gaussian function, we may interpret this result as a Probability Density Function (PDF) that tells us the probability of finding the particle at the point r at time t. Notice that at small t, the probability of finding the particle at $r = 0$ is higher than at any other time. This is the consequence of using the boundary condition given by Equation (4). Figure 1 shows the evolution of C at all one-dimensional points x for different values of t. As we can clearly see, the fact that D is ten times higher on the right graph indicates that it is much easier for the particles of that system to move around.

Now that we have solved the diffusion equation, we may use its solution to obtain the Mean Squared Displacement (MSD) of a trajectory, defined as the squared deviation from the body's initial position over time [3]. To do that, we write the MSD as the second moment[3] of the PDF represented by C in Equation (6). The MSD is mathematically defined as:

$$\langle \vec{r}^2(t) \rangle = \int_{-\infty}^{\infty} r^2 C(r,t) \mathrm{d}\vec{r}. \tag{7}$$

Once we compute the integral in Equation (7), we get

$$\langle \vec{r}^2(t) \rangle = 2nDt. \tag{8}$$

The diffusion modeled by Equation (8) is called **Normal diffusion**. According to [51], Normal diffusion is not the only motion type observed in nature. There are infact four basic motion types: Normal, Anomalous, Direct motion with diffusion and Confined diffusion. Each type has its unique relation with the

[2] If we had not set $M = 1$ in Equation (4), we would have obtained M itself as the result of the integration in Equation (5).

[3] A distribution may be represented by its four moments. The first indicates the central tendency we call the mean. The second is the variance, the third is the skewness that measures the asymmetry of the distribution and the fourth is the kurtosis, a measure of the tails of the distribution [1].

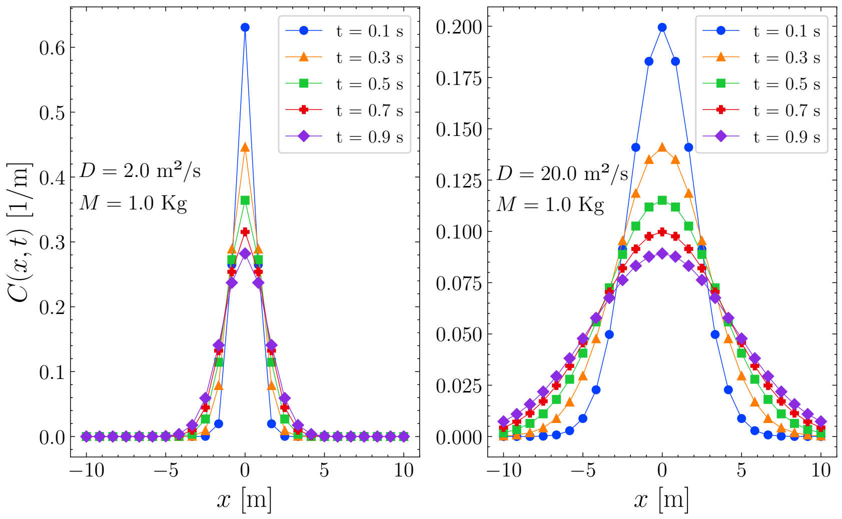

Figure 1: This figure shows the evolution of the concentration C over space and time for different values of the Diffusion Coefficient D. To generate these graphs, we have used Equation (6) and set $M = 1$ Kg for both. In the left graph, we have set $D = 2.0\ \mathrm{m}^2 \cdot \mathrm{s}^{-1}$ and in the right one, $D = 20.0\ \mathrm{m}^2 \cdot \mathrm{s}^{-1}$.

MSD and we can use this relations to extract pieces of information such as the diffusion itself. The mathematical formulations for the different motion types are the following:

$$\langle \vec{r}^2(t) \rangle \propto t, \quad \text{Normal diffusion,} \tag{9}$$

$$\langle \vec{r}^2(t) \rangle \propto t^{\beta}, \quad \text{Anomalous diffusion,} \tag{10}$$

$$\langle \vec{r}^2(t) \rangle \propto t^2, \quad \text{Direct motion with diffusion,} \tag{11}$$

$$\langle \vec{r}^2(t) \rangle \simeq r_c^2[1 - A_1 \exp{(-2A_2nDt/r_c^2)}], \quad \text{Confined diffusion.} \tag{12}$$

In Equation (10), β assumes values less than one, according to [51]. In Equation (12), r_c, A_1 and A_2 are the radius of confinement and two constants that characterize the shape of the confinement, respectively. In addition, we could write Equation (12) as $\langle \vec{r}^2(t) \rangle \propto t^{\beta}$ in order to express the time dependency of the MSD directly. Figure 2 shows a comparison of the four theoretical models for the MSD according to the motion type.

The linear time dependency of the MSD observed in Equation (9) was first calculated by [17], when providing a mathematical description of the motion we nowadays refer to as Brownian motion. The concept of Brownian motion was named after the botanist Robert Brown, who was the first person to observe the irregular motion of particles of polen generated by collisions with particles when immersed in water [23]. In order to mimic such behavior, the Random Walker, a particle that may take multiple steps of random length in a random

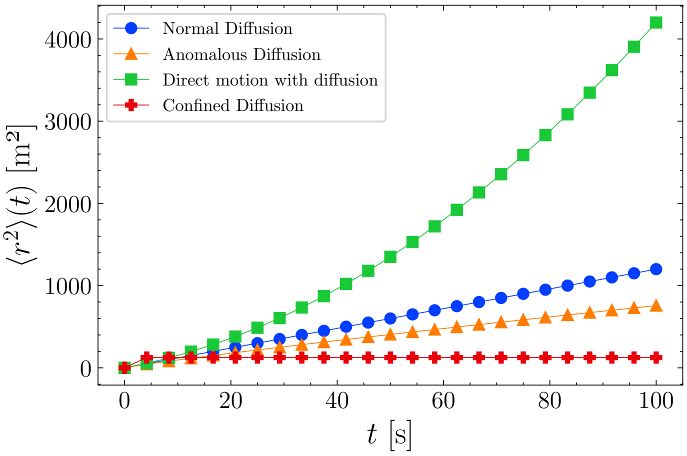

Figure 2: A comparative plot of the four motion types. We defined the numerical values for each parameter as the following: $D = 2.0$, $n = 2$, $v = 0.3$, $A_1 = 2.0$, $A_2 = 3.0$, $r_c = 10.0$ and $\beta = 0.9$. As expected, the MSD for the Confined motion (red line) achieves a plateau, since the motion itself is limited (this will become clear later on). The Direct motion generates a MSD (green line) that grows fast for its quadratic time dependency, whereas the MSD for Normal diffusion (blue line) produces a straight line since the relation between the MSD and time is linear. Given that the exponent β must be less than 1, the MSD for this motion will always be smaller than either Normal diffusion or Direct motion with diffusion.

direction was proposed [13]. We will interpret, from now on, Normal diffusion as the motion type for the Random Walker.

Let us discuss the meaning of Anomalous diffusion. If we recall the two assumptions we made in order to obtain Fick's Laws - where we have stated that the substance in question will avoid any crowded location and that matter is conserved, we considered the environment the substance is located in to be structureless. We made this consideration in an indirect way, since we have not mentioned any details about the structure of the environment.

However, if we do consider the environment to impose geometric constraints of some kind, the first assumption we just mentioned becomes obscure, once we can no longer be sure that the substance will avoid crowded locations. These geometric constraints are one of the reasons we observe Anomalous Diffusion [49]. This behavior may also present itself in many systems, such as Brownian Motion in inhomogeneous systems [43].

An alternative way of describing the rise of Anomalous Diffusion would be to consider systems where the steps a Random Walker takes each time are not independent from each other. Systems as the ones we just described are observed, for instance, in Biology [33].

We mentioned that β in Equation 10 is less than one. This fact was true in the context of study of [51]. However, as a general rule β assumes values that may be smaller or larger than one [42]. When $\beta < 1$, the diffusion regime is called Subdiffusion. On the other hand, if $1 < \beta < 2$, the regime is refered to as Superdiffusion.

Subdiffusion is observed when the movement of particles in a system is slower, on average, than the one observed in Normal Diffusion. This implies that the values of the MSD for Subdiffusion are smaller across time. One interesting example of Anomalous Diffusion in the Subdiffusion regime is the one of molecules' diffusion through the plasma membrane of the human cell [34]. This membrane in known for its crowded inner structure that causes restriction of the motion of macromolecules.

Superdiffusion, on the other hand, happens when the movement of particles is faster, on average, than the one observed in Normal Diffusion. In constrast with Subdiffusion. the values of the MSD across time are high when compared to the MSD for Normal Diffusion. A famous example of Superdiffusion is encountered in the so called *Lévy* flights, where a Random Walker remains in motion without changing direction for a random amount of time [60]. In the present work, we will use "Anomalous diffusion" as a simple way to refer to both subdiffusion and superdiffusion in the context we just described.

In systems where the MSD presents a quadratic time dependency, the active transport of matter is observed [58]. By active transport we mean that the substance that presents diffusion - perhaps Normal diffusion - is immersed in a fluid that moves over time. The Direct motion with diffusion, sometimes called Ballistic diffusion is observed, for instance, in the movement of bacteria [12]. In the present work, we will use "Direct motion with diffusion" as a simple way to refer to situations where the MSD presents a squared time dependency.

Lastly, let us discuss the Confined diffusion. This motion type is observed when the particles of the substance present Normal diffusion while confined in a certain region [10]. This region may have any geometric shape, as long as it is able to contain the substance trapped. Confined diffusion is observed, for instance, when spherical tracers are put inside Periodic Porous Nanostructures [44].

Over the past decades, multiple computational models have been proposed by researchers in order to improve the overall understanding of motion. Theoretical models that aim to mimic single and multiple cell dynamics in both two dimensions [19] and [25] and three dimensions [11] have been vastly proposed. From the perspective of trajectory analysis of three-dimensional Molecular Dynamics Simulations, two computational models among many are proposed by [39] and [45]. Regarding the analysis of cell interactions, [30] designed a package called CellChat. In the context of Machine Learning being used, [37] developed scTour, a package that uses Deep Learning to model cell dynamics. In addition, the trajectory analysis of experimental data has also been discussed in the literature. For an approach that uses Machine Learning in sets of data generated from hand tremors related to Parkinson's Disease, see [47]. Lastly, [56] utilizes Machine Learning in combination with trajectory analysis and ob-

ject tracking techniques in order to model single cell dynamics and diffusion classification.

4 Trajectory quantification

This supplementary material outlines all the physical and statistical measures available in Trajpy for trajectory analysis. Initially, we will explore key physical concepts before delving into the statistical measures. Additionally, we will offer concise descriptions of TrajPy's features and their computational methods.

The first attribute we will discuss is the mean squared displacement (MSD), defined as the deviation of a particle's position with respect to a fixed starting point. There are two mathematically equivalent ways to compute the MSD, according to the Ergodic Hypothesis [3]. We can either compute the MSD by Time Average or Ensemble Average[4] where the former is the usual time average of the position of a single trajectory and the latter is an average of the position taken over the total number of single particle trajectories.

The **MSD by Ensemble Average** is defined as

$$\langle \vec{r}^2 \rangle(t) = \frac{1}{N} \sum_{n=1}^{N} |\vec{r}_n(t) - \vec{r}_n(0)|^2, \tag{13}$$

where N is the total number of trajectories, $\vec{r}_n(t)$ is the position of the n-th particle at time t and $\vec{r}_n(0)$ is the initial position of the same particle. In order to compute this feature, we take the difference between the position of the n-th particle at time t and its initial position. Then we square that difference so that the positive and negative values do not cancel out and move on to the next particle. Once we have squared all the differences using the same value for t, we add all of them and divide the result by the total number of particles N. This is the result for the MSD by Ensemble average for that value of t. The next step is similar to the previous one, but now we use the next available value for t and so on.

The **MSD by Time average** differs from the previous feature due to the fact that it takes in as input a single trajectory. The MSD by Time average is defined by [56] as

$$\langle \vec{r}^2_\tau \rangle = \frac{1}{T-\tau} \sum_{t=1}^{T-\tau} |\vec{r}(t+\tau) - \vec{r}(t)|^2, \tag{14}$$

where τ is the time interval[5], also called the time lag, between the two positions

[4] An ensemble of particles can be interpreted in two ways: a set of particles that differ in their initial positions but are under the same influences in the system or, more generally, repeating the same experiment multiple times where, in each time, the particles present the same initial positions.

[5] When dealing with a trajectory formed by discrete data points - which is the case for TrajPy, the values for τ must, by definition, be a non-negative integer. Negative values would make us compute the difference between the previous position and the next, not the other way around (which is what we want). Non-integer values do not make sense in the context of discrete trajectories, i.e, there is no position ata between the first and second positions.

and T is the total trajectory time. To compute this feature, we interpret $\vec{r}(t+\tau)$ as the position of the particle at the $(t+\tau)$-th time step of the trajectory[6]. By setting $\tau = 1$, since $\tau = 0$ would simply yield zero as result, and $t = 1$, we take the difference between the particle's position at the $(1+\tau)$-th and first time steps. Then we square this difference so that positive and negatives values do not cancel out and move on to the next value of t, in this case, $t = 2$. The next step is to plug in $t = 2, 3, \cdots, T - \tau$ in Equation (14), add the results and divide the final result by $T - \tau$. The numerical output we get is the value of the MSD by Time average for $\tau = 1$. To extract information about the particle's displacement, we can set $\tau = 0, 1, 2, \cdots$ (when analysing discrete trajectories) and compute the MSD by Time average for each value of τ. It is worth mentioning that, since we are always working with finite data, we cannot set a value for τ that is bigger than the data itself, given the definition of τ in this context.

The next feature TrajPy provides is the **MSD Ratio**, a feature that allows the user to characterize the shape of the MSD curve by using two different values for the time lag τ of the MSD by Time Average. The MSD Ratio is defined as:

$$\langle \vec{r}^2 \rangle_{\tau_1,\tau_2} = \frac{\langle \vec{r}^2_{\tau_1} \rangle}{\langle \vec{r}^2_{\tau_2} \rangle} - \frac{\tau_1}{\tau_2}, \tag{15}$$

where $\tau_1 < \tau_2$ and $\langle \vec{r}^2_{\tau_i} \rangle$ is the Mean Squared Displacement by Time average. When we insert Equations (9) - (12) in Equation (15), we see that the MSD Ratio has the following values for each of the four motion types [56]:

$$\begin{cases} \text{MSD Ratio} = 0 & \text{for} \quad \text{Normal diffusion,} \\ \text{MSD Ratio} > 0 & \text{for} \quad \text{Confined and Anomalous diffusion,} \\ \text{MSD Ratio} < 0 & \text{for} \quad \text{Direct motion with diffusion.} \end{cases}$$

That tells us that we can obtain the motion type simply by computing the MSD Ratio or looking at the motion graph. However, if we want to calculate the diffusion constant itself, we may extract this information by either fitting a MSD curve through our data, as we have already established, or computing the so called Green-Kubo's relation for diffusion [35].

Now that we have established the four basic motion types and discussed the importance of the MSD Ratio, we may present the **Anomalous exponent** feature present in TrajPy. We have seen that the time dependency plays an important role in the MSD, so we would expect that the exponent who dictates this dependency is also crucial.

Let us consider the relation between the MSD and the Anomalous diffusion provided by Equation (10):

$$\langle \vec{r}^2 \rangle(t) = 2Dnt^{\beta}.$$

To compute the exponent β, we apply the logarithm function to both sides of this equation and make use of the logarithmic rules to obtain:

$$\log(\langle \vec{r}^2 \rangle(t)) = \log(2nD) + \beta \log(t). \tag{16}$$

[6]Keep in mind that a trajectory is a list of positions with regular time steps, so $t = 1$ means the first time step and so on.

Then, we take the partial derivative with respect to $\log(t)$ on both sides of Equation (16) to end up with:

$$\beta = \frac{\partial \log(\langle \vec{r}^2 \rangle(t))}{\partial \log(t)}, \tag{17}$$

since $\log(2nD)$ does not change with time[7]. Equation (17) provides a simple way to compute the exponent β, we must simply calculate the angular coefficient of the straight line in a log-log graph of the MSD as a function of time.

Figure 3 is a log-log plot of the MSD by Time Average as a function of τ. To construct this plot, we used the log of Equation (10) and set the possible parameters as follows: $D = 2.0$, $n = 2$, $\beta = 0.9$. We can easily check that the angular coefficient of the straight line if Figure 3 is precisely equal to 0.9, so Equation (17) does in fact provide the correct answer.

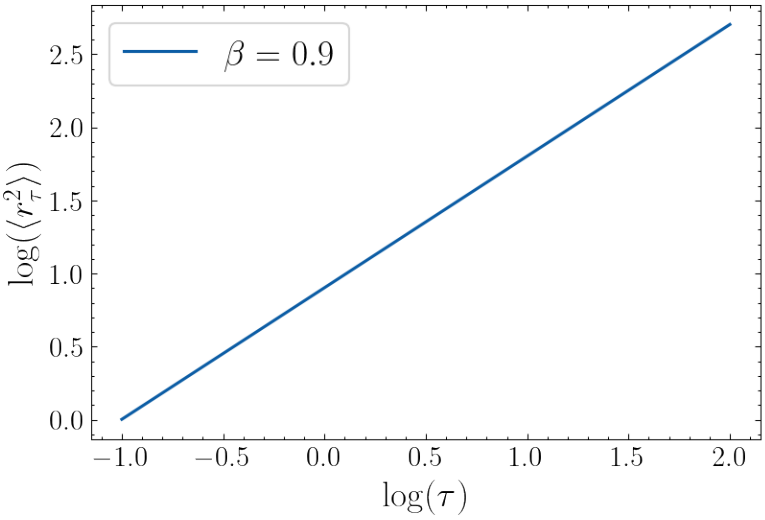

Figure 3: A log-log graph of the MSD by Time Average as a function of τ. The angular coefficient of the straight line produced is the value of the anomalous exponent β.

Another useful feature in TrajPy is the **Fractal Dimension**. This concept is connected to [32] and the idea of Fractal Geometry. Fractals are useful in many fields of study, including medicine [59], social sciences [5] and more. For a complete discussion about the use of fractals emerging from chaos and their applications in Science, see [53].

In TrajPy, we compute the Fractal Dimension D_f of curves proposed by [31], a direct adaptation of the same concept in the Fractal Geometry context to

[7]The diffusion coefficient D may fluctuate around a fixed value over time, but these fluctuations are minimal so we may not consider them.

the analysis of curves in a plane. The Fractal Dimension that interests us is mathematically defined as

$$D_f = \frac{\log n}{\log(ndL^{-1})}, \tag{18}$$

where n is the number of steps taken to form the curve, d is the largest distance between two points of the curve and L is the total length of the curve.

In a sense, D_f tells us how irregular the trajectory is. If the curve is a straight line, then $D_f = 1$ - wich means the trajectory is not irregular at all. However, if $D_f \to 2$, the trajectory can be associated to Brownian motion modeled by Normal Diffusion. And, finally, if a curve is erratic to the point where the motion described by the curve is undergoing some physical limitation - as in the Anomalous or Confined diffusion, for instance - the Fractal Dimension takes values of $D_f > 2$.

Figure 4 shows the value of the Fractal Dimension for each of the four motion types: Normal diffusion, Confined Diffusion, Anomalous diffusion and Direct motion with diffusion. As we can clearly see, the Direct motion with diffusion represented by the green line is a straight line and has a Fractal Dimension of $D_f = 1$. The other three motion types have a Fractal Dimension $D_f > 1$ and the more erratic the motion is, the higher the value of D_f. In addition, the more the curve crosses over itself, the higher the value of D_f.

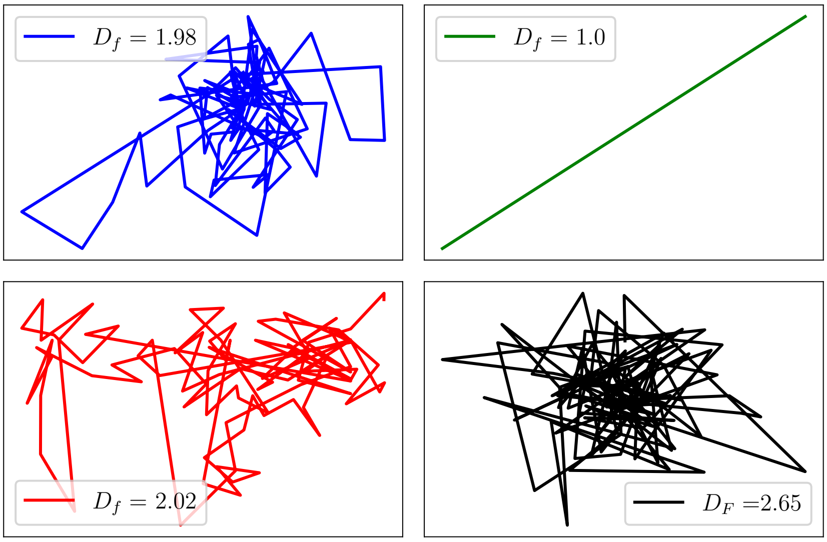

Figure 4: A comparison for different values of the Fractal Dimension D. The blue, green, red and black lines refer to the four basic motion types: Confined Diffusion, Direct motion with diffusion, Normal Diffusion and Anomalous Diffusion.

The next feature we will discuss is the **Radius of Gyration Tensor**, sometimes called Gyration Tensor. There is a close relation between this tensor and the Moment of Inertia Tensor, where both can be used to describe the shape of an object using its distribution of mass and particles. While the Inertia Tensor makes use of the masses of each particle, the Gyration Tensor uses the particles' positions as input to describe the macroscopic object [55].

To describe a distribution of mass, for example, the Gyration Tensor makes use of its eigenvalues, where the approximate shape is obtained from ensembles averages of each eigenvalue of the tensor in question [46]. This is where the Gyration Tensor becomes very useful, since we can use its eigenvalues to describe the shape of the trajectory used in TrajPy and obtain certain properties such as the asymmetry and kurtosis of the trajectory.

The Gyration Tensor $\overleftrightarrow{T}$ is defined as

$$\overleftrightarrow{T} = \begin{pmatrix} R_{xx} & R_{xy} & R_{xz} \\ R_{yx} & R_{yy} & R_{yz} \\ R_{zx} & R_{zy} & R_{zz}, \end{pmatrix} \tag{19}$$

where each element, according to [54], is defined by

$$R_{ij} = \frac{1}{N}\sum_{k=1}^{N} x_i^{(k)} x_j^{(k)} - \frac{1}{N^2}\sum_{k=1}^{N} x_i^{(k)} \sum_{k=1}^{N} x_j^{(k)}. \tag{20}$$

Computing the eigenvalues of the gyration tensor is very useful in many fields of study such as polymer physics, where the Gyration Tensor can describe the dimensions of a polymer chain [21].

Once we compute the eigenvalues and eigenvectors of the diagonalized Gyration Tensor, we may proceed to the next features implemented in TrajPy. The **asymmetry** makes use of R_i, which is the square root of the i-th eigenvalue λ, in such a way that it allows us to verify if there is any tendency for a preferred direction. This means we can analyse any given trajectory and quantify the amount of symmetry or lack thereof across the entire movement. The **asymmetry** was first mathematically defined by [28] as

$$\gamma = -\log\left(1 - \frac{(R_1^2 - R_2^2)^2 + (R_1^2 - R_3^2)^2 + (R_2^2 - R_3^3)^2}{2(R_1^2 + R_2^2 + R_3^2)^2}\right). \tag{21}$$

Given the already established meaning of asymmetry, we can use this concept to evaluate the shape of the trajectory curve. A perfectly symmetric trajectory would yield $\gamma = 0$ whereas a trajectory in a straight line along the main axis would be completely asymmetric - since a straight line is formed by consecutive steps in the same direction - and, therefore, $\gamma \to \infty$. Figure 5 shows a direct comparison of different trajectories and their respective values for γ.

The **Anisotropy** makes use of the eigenvalues of the Gyration Tensor. Defined by [41] as

$$k^2 = 1 - 3\frac{\lambda_1\lambda 2 + \lambda_2\lambda_3 + \lambda_3\lambda_1}{(\lambda_1 + \lambda_2 + \lambda_3)^2}, \tag{22}$$

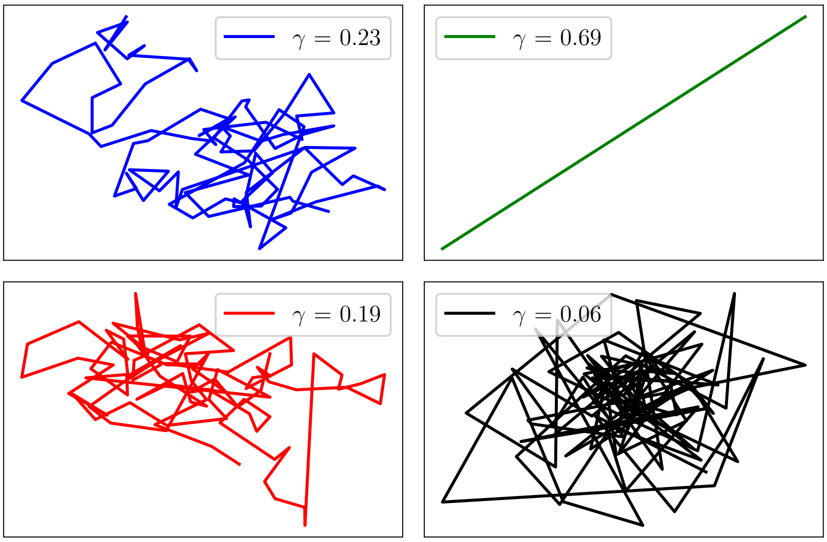

Figure 5: This image shows a direct comparison for different values of the asymmetry γ. The blue, green, black and red lines refer to the four basic motion types: Confined diffusion, Direct motion with diffusion, Anomalous diffusion and Normal diffusion. As expected, the straight green line provides the highest value of γ.

the anisotropy contains information about the symmetry and dimensionality of the system. In simple terms, the anisotropy is the characteristic of a physical property that changes its value according to the direction it is measured [14]. One simple example of anisotropy would be the mass anisotropy, where the amount of matter changes according to the direction we look. In Equation (22), k^2 is limited to values between 0 and 1 where $k^2 = 0$ indicates the distribution of positions is symmetric in relation to the origin and, therefore, all eigenvalues are the same. If $k^2 = 1$, that same distribution is not symmetric in relation to the origin and implies that at least two eigenvalues are zero. This happens because the positions may be aligned as a linear chain and and there is only one eigenvector that could represent it [21]. Figure 6 provides a comparison of the anisotropy for four different trajectories.

Another simple and yet useful quantity provided by TrajPy is the **Straightness** of the trajectory. The straightness measures the similarity between the actual trajectory and a straight line. This quantity, according to [6], is defined as

$$S = \frac{|\vec{r}_{N-1} - \vec{r}_0|}{\sum_{i=1}^{N-1} |\vec{r}_i - \vec{r}_{i-1}|}, \tag{23}$$

where the numerator is the distance, measured as a straight line, between the

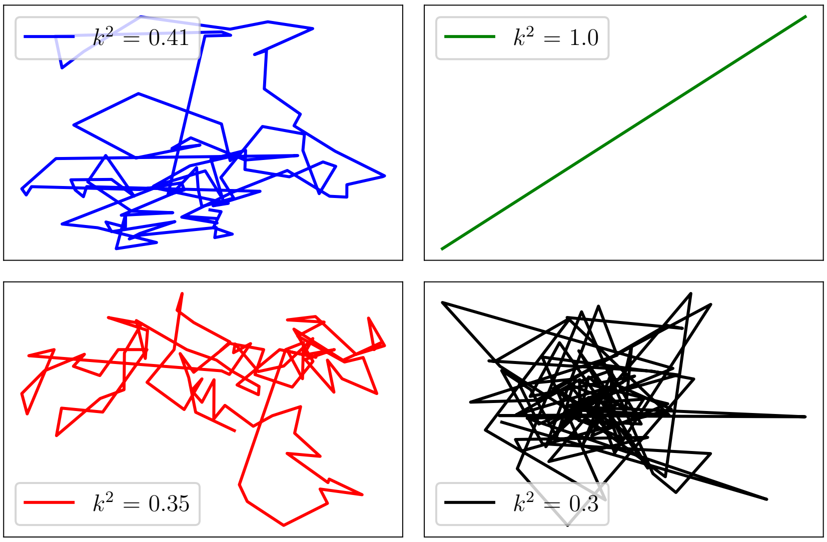

Figure 6: This image shows a direct comparison for different values of the anisotropy k^2. The blue, green, black and red lines refer to the four basic motion types: Confined diffusion, Direct motion with diffusion, Anomalous diffusion and Normal diffusion. As expected, the straight green line provides the highest value of k^2.

initial and second to last positions $\vec{r}_0$ and $\vec{r}_{N-1}$ respectively. The denominator is the length of the trajectory measured as the sum of the individual distances between each pair of points of the trajectory.

If the trajectory takes the form of a straight line, then both terms of the fraction in Equation (23) are equal and, therefore, $S = 1$. However, as the trajectory gets less similar to a straight line, the larger is the length of the trajectory and, by extension, the larger the denominator becomes. In such cases, $S \approx 0$ as the trajectory increases in length. Figure 7 shows a comparison of different trajectories and their respective values of S.

The **efficiency** of the trajectory, another quantity present in TrajPy, relies on a similar principle that of the **straightness**. Mathematically defined as

$$E_{ff} = \frac{|\vec{r}_{N-1} - \vec{r}_0|^2}{\sum_{i=1}^{N-1} |\vec{r}_i - \vec{r}_{i-1}|^2}, \tag{24}$$

the **efficiency** relates the square of the net displacement with the square of the trajectory length. According to [56], if the particle's initial and final positions r_0 and r_{N-1} are the same, the **efficiency** $E_{ff} = 0$ for any trajectory length. On the other hand, for a given net displacement - the numerator in Equation (24) - the more irregular the trajectory is, the smaller the value for E_{ff} in relation to a

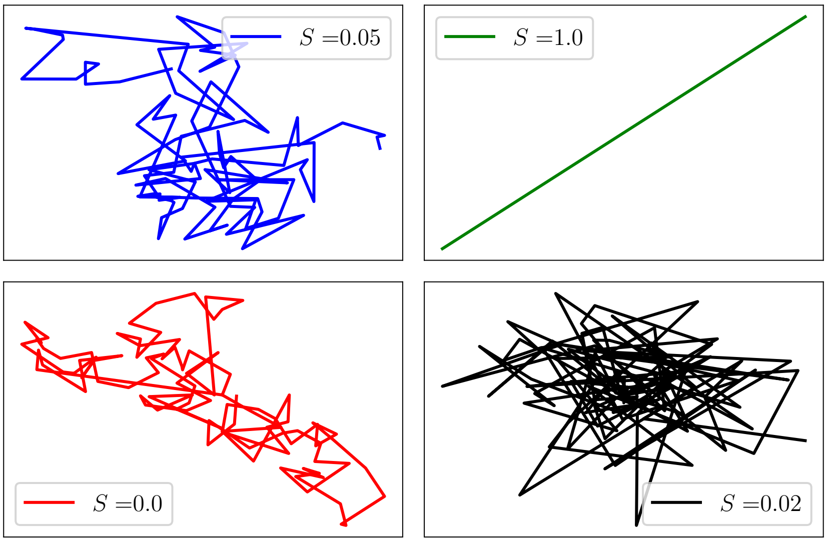

Figure 7: This image shows a direct comparison for different values of the straightness S. The blue, green, black and red lines refer to trajectories presenting the four basic diffusion types: Confined diffusion, Direct motion with diffusion, Anomalous diffusion and Normal diffusion. As we expected, the straight green line presents $S = 1$.

straight line. Figure 8 shows a comparison for the values of E_{ff} for trajectories of different diffusion types.

The next quantity present in TrajPy is the **gaussianity** of the trajectory. Simply put, this quantity measures how similar to a Gaussian Distribution the trajectory is. The **gaussianity** is defined by [18] as

$$G(\tau) = \frac{2\langle \vec{r}_\tau^4 \rangle}{3\langle \vec{r}_\tau^2 \rangle^2} - 1, \tag{25}$$

where $\langle r_\tau^n \rangle$ is the n-th moment of the distribution computed by

$$\langle \vec{r}_\tau^n \rangle = \frac{1}{T - \tau} \sum_{t=1}^{T-\tau} |\vec{r}(t+\tau) - \vec{r}(t)|^n. \tag{26}$$

In Equation (26), T is the total number of steps and τ is the time lag. The **gaussianity** is useful when one wants to have an idea about the kind of diffusion a specific particle presents. For normal diffusion, we expect G to be zero and for the other diffusion types, G will present values different than zero [56]. Figure 9 shows a direct comparison for values of the **gaussianity** for different diffusion kinds.

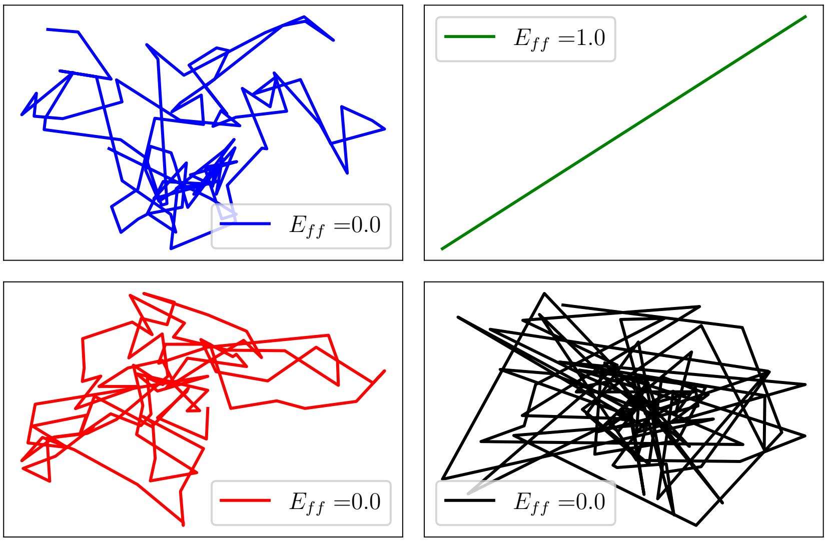

Figure 8: This image shows a comparison for different values of the efficiency E_{eff}. The blue, green, black and red lines refer to trajectories presenting the four basic diffusion types: Confined diffusion, Direct motion with diffusion, Anomalous diffusion and Normal diffusion. As we can see, E_{ff} achieves the highest value for a straight line and rapdly goes to zero as the trajectory becomes more irregular.

The next feature we will discuss is the **kurtosis**. It measures the tailedness of the distribution of positions once we project this distribution along the eigenvector corresponding to the highest eigenvalue or the **gyration tensor** [26]. To compute this feature, we take the scalar product between each position $\vec{r}_i$ and the main eigenvector $\vec{e}_1$: $r_i^p = \vec{r}_i \cdot \vec{e}_1$ and calculate the quartic moment of r_i^p given by

$$K = \frac{1}{N} \sum_{i=1}^{N} \frac{(r_i^p - \langle r^p \rangle)^4}{\sigma_{r^p}^4}. \tag{27}$$

In equation (27), $\langle r^p \rangle$ is the mean position of the projected trajectory and $\sigma_{r^p}^4 = (\sigma_{r^p}^2)^2$ is the variance of r_i^p. To put it in simple terms, the **kurtosis** measures how long the tails of the position distribution are, it deals with the outliers - the data points far away from the expected value we usually call the mean. The higher the value of the **kurtosis**, the longer the tails are, and, by extension, the larger the number of outliers. Figure 10 provides a comparison for the values of kurtosis of four different trajectories when projected along their corresponding main eigenvector.

Given that, in TrajPy, we analyse trajectories that are composed of discrete data points, we compute the approximate velocity as the ratio between the

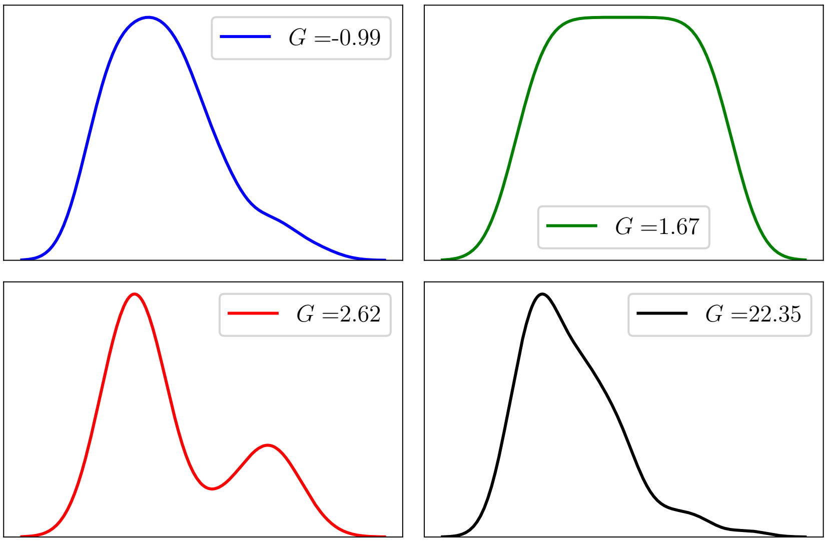

Figure 9: A comparison for different values of the efficiency G. The blue, green, black and red lines refer to trajectories presenting the four basic diffusion types: Confined diffusion, Direct motion with diffusion, Anomalous diffusion and Normal diffusion.

displacement $\vec{r}_{i+1} - \vec{r}_i$ and the time step $t_{i+1} - t_i$.

The **Velocity Autocorrelation Function** (VACF) is a quantity that measures the correlation between velocities at different times [2]. According to [16], the VACF is defined as

$$C_v(t) = \frac{1}{N}\sum_{i=1}^{N}(\vec{v}_i(t) \cdot \vec{v}_i(t=0)) = \langle v(t)v(0) \rangle, \tag{28}$$

where $\vec{v}_i(t)$ is the velocity of the i-th particle at time t and N is the total number of particles of the system. If we were to describe Equation (28) in simple terms, we could say that we take the scalar product of the velocity vectors of the particle i at different times in order to calculate how similar both vectors are[8].

We care about this similarity - this **correlation** - because the way the velocity vectors differ from one another gives us information about both the system's thermodynamical equilibrium and dynamics. Which means the **Velocity Autocorrelation Function** contains information about the dynamical nature of the molecular process [36].

For forces with different magnitudes applied to different systems, such as solids and liquids, the VACF presents different behaviors. For instance, we would expect it to be a straight line if there are no forces acting on the system,

[8]Notice that we compute the scalar product for different velocities for all N particles.

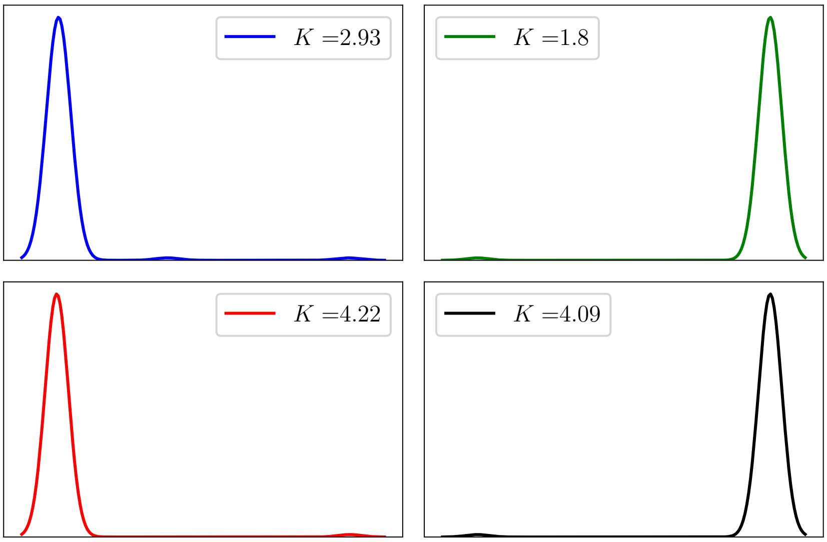

Figure 10: This image shows a comparison for different values of the efficiency K. The blue, green, black and red lines refer to trajectories presenting the four basic diffusion types: Confined diffusion, Direct motion with diffusion, Anomalous diffusion and Normal diffusion when projected along their corresponding main eigenvector. The longer the tails, the larger the value of the kurtosis K.

since the momentum of each particle would remain the same over time. On the other hand, for weak forces, the VACF decays exponentially over time because a weak force gradually changes the momentum of the particles and, by extension their velocities became gradually different[9]. For strong forces we may observe a few - or even several - oscillations[10] of the VACF between positive and negative values. This happens because strong forces make the particles seek the position where the net force is as small as possible. Once the particles reach such state, they oscilate back and forth around this equilibrium and these oscilations produce the oscilations in the VACF [57]. Figure 11 provides a comparison for the behavior of the VACF for $t = 100$ time steps for four different trajectories.

As we can see in Figure 11, three of the four trajectories's velocities' decorrelate over time whereas the green trajectory does precisely the opposite. This is observed because the trajectory of the particle with Direct motion with diffusion was simulated in such a way that its velocities are dependent of one another.

We can make use of the VACF to discuss the next feature present in TrajPy, the **Green-Kubo Relation** for the diffusion coefficient D. According to [22], this

[9]In Statistical Mechanics, we say the velocity **decorrelates** over time.

[10]These oscillations are very similar to the ones we may observe in a Damped Harmonic Oscilator.

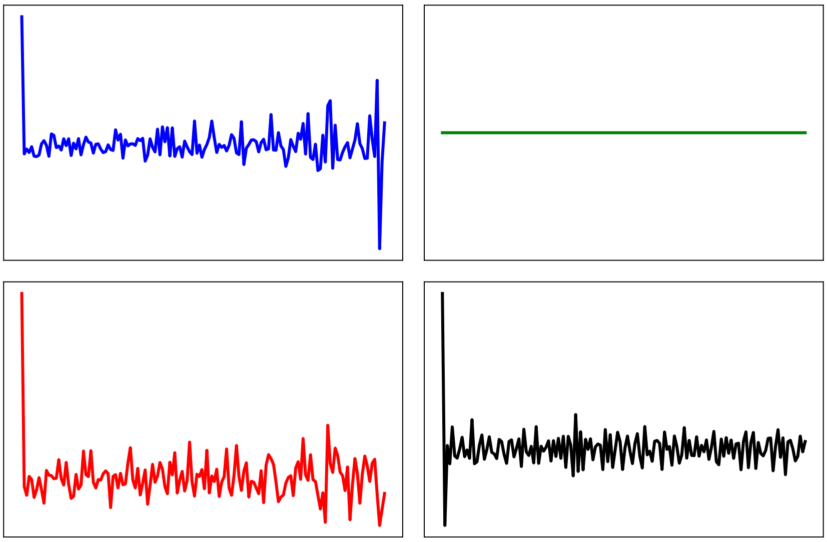

Figure 11: This image shows a comparison for different behavior of the Velocity Autocorrelation Function. The blue, green, black and red lines refer to trajectories presenting the four basic diffusion types: Confined diffusion, Direct motion with diffusion, Anomalous diffusion and Normal diffusion.

relation is defined as

$$D = \int_0^\infty d\tau \langle v(\tau)v(0)\rangle, \tag{29}$$

where $\langle v(\tau)v(0)\rangle$ is the VACF.

Equation (29) is the **Green-Kubo Relation** for the diffusion coefficient D. It connects the macroscopic concept of diffusion with the microscopic movement of the particles. There are Green-Kubo relations for multiple **transport coefficients**, such as diffusion. Simply put, transport coefficients measure how quickly a perturbed system returns to equilibrium [3]. So in the case of the diffusion D, the system might be in equilibrium and a perturbation such as a drop of ink in a glass of water will make the particles of the system rearrange themselves so that the equilibrium is once again achieved[11].

The next feature recently implemented in TrajPy is a **statistical description of the velocity**. This feature takes as input the values of the velocity of each particle over time and computes the mean, median, mode, variance, standard deviation, the distance between the largest and smallest value of the velocity (called range), the kurtosis and skewness of the distribution - for a complete discussion about these concepts and the equations we are about to provide,

[11] The movement of particles towards the opposite direction of the concentration gradient described in Subsection 3 is a general way to say that the system will always seek equilibrium.

see [1]. Although these calculations are quite simple, they allow us to describe the velocity distribution using some basic concepts from Statistics.

The mean $\langle v^{(i)} \rangle$ of the velocity is the usual expected value defined as

$$\langle v^{(i)} \rangle = \frac{1}{n} \sum_{k=1}^{n} v_k^{(i)}, \tag{30}$$

where n is the total number of data points[12] for the velocity, $i = x, y, z$ and k indicates the k-th value of the velocity. From this point, we will ommit the vector notation for simplicity.

The median is the data point where one half of the data points lies above it and the other half below it, when put in ascending order. The median $m_v^{(i)}$ of the velocity is mathematically defined as

$$m_v^{(i)} = \begin{cases} v_{\frac{n+1}{2}}^{(i)} & \text{if} \quad n \text{ is odd,} \\ \left[v_{\frac{n}{2}}^{(i)} + v_{\frac{n}{2}+1}^{(i)} \right] /2 & \text{if} \quad n \text{ is even.} \end{cases} \tag{31}$$

where $v_{(n+1)/2}^{(i)}$ is the $(n + 1)/2$-th velocity data point in the i-th direction.

The mode is the velocity value that is repeated the most along the list of values for the velocity in each direction- in cases where there is more than one mode, we consider all of them. To compute this quantity, we used a Python module called Statsmodels [52].

The velocity variance $var_v^{(i)}$ describes the average squared distance between the velocity data points and the mean $\langle v^{(i)} \rangle$ and is mathematically defined as

$$var_v^{(i)} = \frac{1}{n} \sum_{k=1}^{n} (v_k^{(i)} - \langle v^{(i)} \rangle)^2. \tag{32}$$

The standard deviation $std_v^{(i)}$ of the velocity, on the other hand, is the square root of the variance so that the average distance described in Equation (32) can be written in the same units as the velocity itself. The standard deviation calculated for the velocity data points in the i-th direction is defined as

$$std_v^{(i)} = \sqrt{var_v^{(i)}}. \tag{33}$$

The range of the velocity $ran_v^{(i)}$ is defined to be the absolute value of the difference between the largest and the lowest observed values for the velocity in the i-th direction. It is simply defined as

$$ran_v^{(i)} = |max_v^{(i)} - min_v^{(i)}|. \tag{34}$$

The skewness $S^{(i)}$ measures the lack of symmetry of the velocity probability distribution function when the center point is considered. The more different

[12] In this case, we are considering that the velocity is composed of discrete data points.

the left and right sides of the distribution are, the higher the skewness. For a Gaussian distribution, the skewness is null, since perfect symmetry is observed. In the case of a distribution with a short left tail and a long right one, the skewness will be positive. If the opposite is observed, the skewness will be negative. The skewness is defined as

$$S^{(i)} = \frac{(var_v^{(i)})^{3/2}}{std_v^{(i)}}. \tag{35}$$

The kurtosis measures the shape of the peak and tails of the velocity probability distribution function. In TrajPy, we use what is called excess kurtosis, where the calculations are done in such a way that a Gaussian Distribution has a kurtosis of zero - hence the value of three being subtracted in Equation (36). When looking at a distribution, if its peak is sharper and its tails contain more data then the ones of a Normal Distribution, the kurtosis will have a positive value. On the other hand, if the distribution presents a flat peak with long tails, its associated kurtosis will be negative. The excess kurtosis $K^{(i)}$ is defind as

$$K^{(i)} = \frac{(var_v^{(i)})^{3/2}}{(std_v^{(i)})^4} - 3. \tag{36}$$

Figure 12 provides an illustration for the behavior of probabilities distribution functions and their respective values for the skewness and kurtosis. Notice that both of these measurements give us insight about the overall behavior of the velocity.

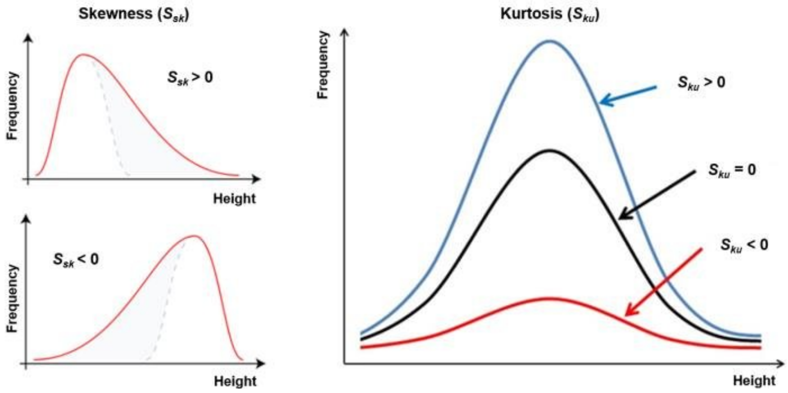

Figure 12: This figure provides the possible outcomes for both the skewness and kurtosis of the intensity - here refered to as "height" - distribution function of pixels in a topographic image. The velocity distribution function follows the same idea regarding the values of kurtosis and skewness. Adapted from [9].

The last attribute implemented in TrajPy is the **frequency spectrum** performed by the Fourier Transform - for a thorough discussion regarding the

Fourier Transform, see [4]. The integral transformation called Fourier Transform is one of the many of its kind and produces relevant information about any function $f(t)$, as long as the function is differentiable everywhere in its time domain. The Fourier Transform is defined as

$$g(\omega) = \frac{1}{\sqrt{2\pi}} \int_{-\infty}^{\infty} f(t) e^{i\omega t} dt, \tag{37}$$

where $g(\omega)$ is the analogous of $f(t)$ in the frequency domain ω.

We are particularly interested in the fact that the Fourier Transform reveals what are the trigonometric functions $\sin(t)$ and $\cos(t)$ present in $f(t)$ if represent such function as linear combinations of both trigonometric functions. For an application of the Fourier Transform in the analysis of hand tremors, see [47].

In TrajPy, we use this linear combination of trigonometric functions to get access to the underlying frequencies of vibration of the system along with its amplitude. To do that, we use the Python numerical library Numpy [24], specifically the Fast Fourier Transform algorithm [40]. By using this mathematical tool, we are able to provide the trajectory of any particle, access the trigonometric linear combination and compute the following attributes: the highest frequency and its associated amplitude, the mean frequency, the frequencies above a given threshold specified by the user along with their amplitudes, the frequency spectrum and amplitudes.

To compute these features, we apply the Fast Fourier Transform algorithm to every data point of the trajectory and store the information. The dominant frequency μ is the highest frequency and, by extension, the dominat amplitude is the amplitude of the wave with frequency μ. The mean frequency is computed by taking the mean of all frequencies, whereas the main frequencies are calculated once we provide a threshold and select the frequencies that are higher than or equal to this threshold along with their corresponding amplitudes. The frequency spectrum is a list of all frequencies calculated for every trajectory data point and the amplitudes are the absolute value of each frequency in the spectrum. We take the absolute value of the frequencies so that we can get the amplitudes. Figure 13 shows an application of the Fast Fourier Transform algorithm.

5 Trajectory simulations

In this section, we will present the way we simulate the four motion types we have discussed in Section 3.

Let us begin by describing the simulation procedure for Normal Diffusion, as proposed by [38]. In order to simulate a particle in ϕ dimensions whose trajectory is modeled by Normal Diffusion, we simulate ϕ one-dimensional brownian motions and return the time steps and associated one-dimensional trajectories. The ϕ one-dimensional brownian motions are then combined as spatial components x, y and z, where each component is scaled to have unitary length.

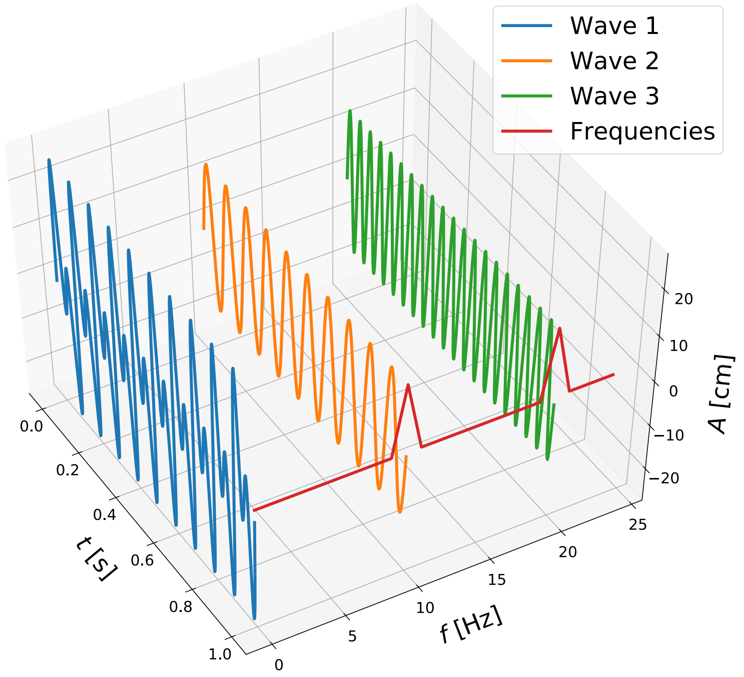

Figure 13: This figure shows a wave (blue) that is formed by the sum of two different waves (green and yellow). The red line presenting two peaks is a visual representation of the effects of applying the Fast Fourier Transform algorithm to the blue wave, once the units of measurement of the red line is Hz, the unit of frequency. The corresponding amplitudes of each wave can be verified by looking at the Amplitude axis.

In simple terms, for each of the one-dimensional brownian motions, we draw a random number a out of a standard Gaussian distribution with unitary mean and standard deviation. Then we set $u = (0.5 - a)dx$, where dx is the maximum step length the particle is allowed to take in any direction, and check whether a is greater than or equal to the absolute value of $C(u)$, where C is the Concentration we have defined in Equation 6. If any of the previous conditions is true, then the particle takes a step of length u in a random direction. This process is repeated for all the given time steps for all ϕ one-dimensional brownian motions.

As stated in the beginning of this chapter, we may change a set of parameters for each simulation engine for the four basic motion types. In the case of Normal Diffusion, we may change the number of displacements the particle will present,

the number of dimensions[13], the maximum step length dx, the initial position, the diffusion coefficient D and the time step t that will be used in Equation 6.

The next motion type we will discuss is the Confined Diffusion. To simulate it, we specify the number of displacements, the number of dimenions, dx, the initial position, D and the time step. For each displacement out of the total number of displacements determined as a parameter, the particle will present Normal Diffusion behavior for 100 displacements. If the particle, after those 100 displacements, remains inside a sphere whose radius is also defined as a parameter, the new position of the particle will be the position associated with the 100th displacement. Regarding the radius of confinement of the sphere, we suggest using positive values up tp 0.5, since the larger the radius, the more similar to Normal Diffusion the Confinement Diffusion becomes.

The third motion type is the Direct Motion with Diffusion, which is a combination of a simple integration of a constant velocity v over time and Normal Diffusion. The direct motion with diffusion is given by

$$\vec{r}(t) = \vec{\Gamma}(t) + \vec{r}_0 + \int_{t_0}^{T} \vec{v}\mathrm{d}t, \tag{38}$$

where $\vec{\Gamma}(t)$ is the position generated from Normal Diffusion at time t, $\vec{r}_0$ is the initial position of the particle determined as a parameter in the algorithm used to simulate Direct motion with diffusion and $\vec{v}$ is the constant velocity also determined as a parameter. The remaining parameters are the number of displacements, the number of dimensions and the time step.

The combination of Direct Motion and Normal Diffusion yields different behaviors, depending on the magnitude of the velocity v in Equation 38. For $v \approx 0.01$, the overall diffusion resembles a Normal Diffusion. For $v > 1.0$, the same overall diffusion becomes more similar to the Direct Motion modeled by Equation 38. Figure 14 provides a simple comparison of four different trajectories presenting the behavior of Direct Motion with Diffusion for four different values of the velocity v.

In the literature, there are different methods to simulate Anomalous Diffusion, such as obstruction of the particle's path, fractional brownian motion and continuous-time random walk [48]. In TrajPy, we have chosen to simulate this diffusion by means of the Weierstrass-Mandelbrot function[14] [7] for the simplicity associated with its computational calculation.

Put in simple terms, the Weierstrass-Mandelbrot function is continuous in its entire domain, differentiable nowhere and mathematically defined as

$$W(t) = \sum_{n=-\infty}^{\infty} \frac{\cos(\phi_n) - \cos(\gamma^n t^* + \phi_n)}{\gamma^{n\beta/2}}. \tag{39}$$

[13]We may set the number of dimensions as the product between the number of particles we wish to simulate and the number of dimensions for each particle or loop over the number of particles simulating one at a time.

[14]In the present moment, we are solely interested in the practical aspect of such function from a computational perspective. The Weierstrass-Mandelbrot function presents a connection with fractals and fractional browninan motion, but its origins and meaning are beyond the scope of this manuscript.

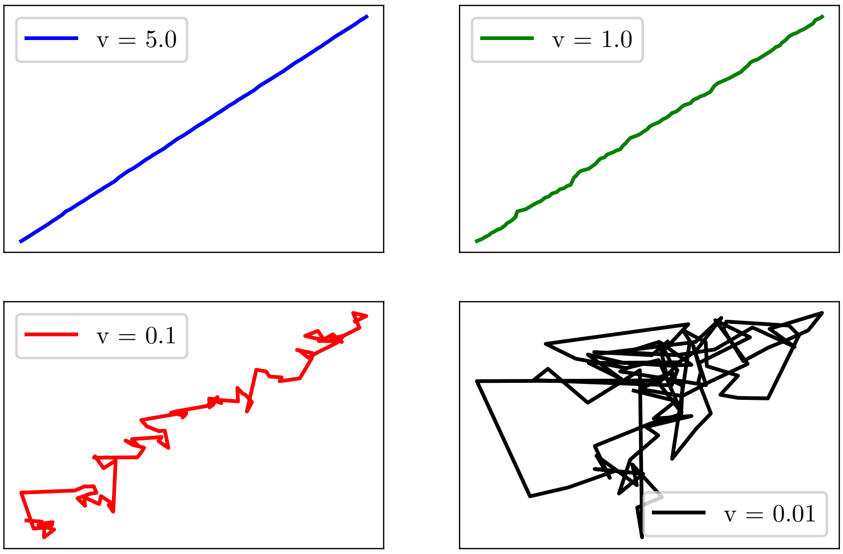

Figure 14: This figure presents the effect of the velocity's magnitude on the overall behavior of the Direct Motion with Diffusion. The smaller the velocity, the more similar to a Random Walker the trajectory becomes.

In Equation 39, ϕ_n is a phase randomly chosen from the interval $[0, 2\pi]$ for every value of the summation index n, $\gamma = \pi^{1/2}$ and $t^* = 2\pi t/N$, where N is the total number os steps the particle may take in a simulation.

Every individual position of the trajectory is obtained by performing the summation in Equation 39 from $n = -8$ to $n = 48$, since, as determined by [50], adding more positive and negative values has little to no effect in the position, i.e., the numerical value of the position does not change much if the summation is performed over a larger interval than $[-8, 48]$ for n.

Regarding the parameters, they are the number of displacements, the number of dimensions, the time step and the exponent β. We advise the user to use values of β within the interval (0,2], where we must exclude the value of $\beta = 1$, since this specific value would yield a Normal Diffusion, as we have discussed previously.

Table S1 – Descriptive table of the attibutes.

Attribute	Measurement	Possible Values
MSD by Time average	Average quadratic displacement	(0,-)
MSD by Ensemble average	Average quadratic displacement	(0,-)
MSD Ratio	Ratio between two MSD's	MSD Ratio = 0 $\rightarrow$ Normal diffusion,<br>Positive MSD Ratio $\rightarrow$ Confined or Anomalous diffusion,<br>Negative MSD Ratio $\rightarrow$ Direct motion with diffusion.
Anomalous Exponent	Time dependency of the MSD	(0,-)
Fractal Dimension	Trajectory's irregularity	Fractal Dimension = 1 $\rightarrow$ Trajectory is a straight line,<br>Fractal Dimension $\approx$ 2 $\rightarrow$ Trajectory resembles a Random Walker,<br>Fractal Dimension$>$ 2 $\rightarrow$ Trajectory undergoes physical limitations.
Gyration Radius	Description of the shape of a curve	(-,-)
Asymmetry	Existence of preferred direction of motion	(0,∞)
Anisotropy	Symmetry of the positions' distribution	(0,1)
Straightness	Similarity between the trajectory and a straight line	(0,1)
Positions' Kurtosis	Shape of the positions' distribution	$K =< 3$ $\rightarrow$ Flat peak and long tails,<br>$K = 3$ $\rightarrow$ Gaussian Distribution,<br>$K => 3$ $\rightarrow$ Sharp peak and short tails.
Gaussianity	Similarity between the positions' distribution and a Gaussian distribution	(0,-)
Efficiency	How effective the trajectory is	(0,1)
Velocity	Displacement per unit of time	(-,-)
Velocity Autocorrelation Function	Similarity of the particle's velocity at different times	(-,-)
Green-Kubo Relation	Diffusion coefficient	(0,-)
Velocity Description	Central tendency and spread of the velocity distribution	(-,-)
Frequency Spectrum	Underlying frequencies via Fourier Transform	(0,-)

References

[1] Basant Lal Agarwal. *Basic Statistics*. New Age International, 2006.

[2] Berni Julian Alder and T. E. Wainwright. "Decay of the Velocity Autocorrelation Function". In: *Physical Review A* 1 (1 1970), pp. 18–21. DOI: 10.1103/PhysRevA.1.18.

[3] Michael P. Allen and Dominic J. Tildesley. *Computer Simulation of Liquids*. USA: Clarendon Press, 1989.

[4] George Arfken. *Mathematical Methods for Physicists*. Third. San Diego: Academic Press Inc., 1985.

[5] Michal Banaszak et al. "Self-Organisation in Spatial Systems-From Fractal Chaos to Regular Patterns and Vice Versa". In: *PloS one* 10.9 (2015).

[6] Simon Benhamou. "How to reliably estimate the tortuosity of an animal's path:: straightness, sinuosity, or fractal dimension?" In: *Journal of Theoretical Biology* 229.2 (2004), pp. 209–220. DOI: https://doi.org/10.1016/j.jtbi.2004.03.016.

[7] Michael Victor Berry, Z. V. Lewis, and John Frederick Nye. "On the Weierstrass-Mandelbrot Fractal Function". In: *Proceedings of the Royal Society of London. Series A, Mathematical and Physical Sciences* 370.1743 (1980), pp. 459–484.

[8] Mary L. Boas. "Mathematical Methods in the Physical Sciences, 2nd ed." In: *American Journal of Physics* 67.2 (1999), pp. 165–169. DOI: 10.1119/1.19218.

[9] Attila Bonyár. "Application of localization factor for the detection of tin oxidation with AFM". In: 2015. DOI: 10.1109/SIITME.2015.7342289.

[10] Poornachandra Sekhar Burada et al. "Diffusion in Confined Geometries". In: *ChemPhysChem* 10 (2009), pp. 45–54. DOI: https://doi.org/10.1002/cphc.200800526.

[11] Eric Campbell and Prosenjit Bagchi. "A computational model of amoeboid cell swimming". In: *Physics of Fluids* 29.10 (2017). DOI: 10.1063/1.4990543.

[12] Avi Caspi, Rony Granek, and Michael Elbaum. "Diffusion and directed motion in cellular transport". In: *Physical Review E* 66 (2002). DOI: 10.1103/PhysRevE.66.011916.

[13] Edward Codling, Michael Plank, and Simon Benhamou. "Random walks in biology". In: *Journal of the Royal Society* 5 (2008), pp. 813–834. DOI: 10.1098/rsif.2008.0014.

[14] Jacques Colin et al. "Evidence for anisotropy of cosmic acceleration". In: *Astronomy and Astrophysics* 631 (2019). DOI: 10.1051/0004-6361/201936373.

[15] Mysore A. Dayananda. “A Direct Derivation of Fick’s Law from Continuity Equation for Interdiffusion in Multicomponent Systems”. In: *Scripta Materialia* 210 (2022). DOI: `https://doi.org/10.1016/j.scriptamat.2021.114430`.

[16] M. A. Despósito and A. D. Viñales. “Subdiffusive behavior in a trapping potential: Mean square displacement and velocity autocorrelation function”. In: *Physical Review E* 80 (2009). DOI: `10.1103/PhysRevE.80.021111`.

[17] Albert Einstein. *Investigations on the Theory of the Brownian Movement*. Courier Corporation, 1956.

[18] Dominique Ernst, Jürgen Köhler, and Matthias Weiss. “Probing the type of anomalous diffusion with single-particle tracking”. In: *Physical Chemistry Chemical Physics* 16.17 (2014), pp. 7686–7691.

[19] Yuqiang Fang et al. “An Active Biomechanical Model of Cell Adhesion Actuated by Intracellular Tensioning-Taxis”. In: *Biophysical Journal* 118 (2020). DOI: `10.1016/j.bpj.2020.04.016`.

[20] Dr. Adolph Fick. “V. On liquid diffusion”. In: *The London, Edinburgh, and Dublin Philosophical Magazine and Journal of Science* 10.63 (1855), pp. 30–39. DOI: `10.1080/14786445508641925`.

[21] Marshall Fixman. “Radius of Gyration of Polymer Chains”. In: *The Journal of Chemical Physics* 36.2 (1962), pp. 306–310. DOI: `10.1063/1.1732501`.

[22] Daan Frenkel and Berend Smit. *Understanding Molecular Simulation: From Algorithms to Applications*. Second. Vol. 1. Computational Science Series. San Diego: Academic Press, 2002.

[23] Arthur Genthon. “The concept of velocity in the history of Brownian motion”. In: *The European Physical Journal H* 45 (2020), pp. 49–105. DOI: `10.1140/epjh/e2020-10009-8`.

[24] Charles R. Harris et al. “Array programming with NumPy”. In: *Nature* 585 (2020), pp. 357–362. DOI: `10.1038/s41586-020-2649-2`.

[25] Tommy Heck et al. “The role of actin protrusion dynamics in cell migration through a degradable viscoelastic extracellular matrix: Insights from a computational model”. In: *PLOS Computational Biology* 16.1 (2020), pp. 1–34. DOI: `10.1371/journal.pcbi.1007250`.

[26] Jo A Helmuth et al. “A novel supervised trajectory segmentation algorithm identifies distinct types of human adenovirus motion in host cells”. In: *Journal of structural biology* 159.3 (2007), pp. 347–358.

[27] Hiromasa Hirakawa et al. “Relationship between Self-Diffusion and Interdiffusion in Gaseous Systems”. In: *Bulletin of the Chemical Society of Japan* 46.9 (1973), pp. 2659–2662. DOI: `10.1246/bcsj.46.2659`.

[28] S'ebastien Huet et al. "Analysis of transient behavior in complex trajectories: application to secretory vesicle dynamics". In: *Biophysical journal* 91.9 (2006), pp. 3542–3559.

[29] Meyer B. Jackson. *Molecular and Cellular Biophysics*. Cambridge University Press, 2006.

[30] Suoqin Jin et al. "Inference and analysis of cell-cell communication using CellChat". In: *Nature Communications* 12 (2021). DOI: 10.1038/s41467-021-21246-9.

[31] Michael J. Katz and Edwin B. George. "Fractals and the analysis of growth paths". In: *Bulletin of Mathematical Biology* 47.2 (1985), pp. 273–286. DOI: https://doi.org/10.1016/S0092-8240(85)90053-9.

[32] M.J. Kirkby. *The fractal geometry of nature*. 1983. DOI: 10.1002/esp.3290080415.

[33] Joseph Klafter and Igor M Sokolov. "Anomalous diffusion spreads its wings". In: *Physics World* 18.8 (Aug. 2005), pp. 29–32. DOI: 10.1088/2058-7058/18/8/33. URL: https://doi.org/10.1088/2058-7058/18/8/33.

[34] Diego Krapf. "Chapter Five - Mechanisms Underlying Anomalous Diffusion in the Plasma Membrane". In: *Lipid Domains*. Ed. by Anne K. Kenworthy. Vol. 75. Current Topics in Membranes. Academic Press, 2015, pp. 167–207. DOI: https://doi.org/10.1016/bs.ctm.2015.03.002.

[35] M. Howard Lee. "Fick's Law, Green-Kubo Formula, and Heisenberg's Equation of Motion". In: *Physical Review Letters* 85 (12 2000), pp. 2422–2425. DOI: 10.1103/PhysRevLett.85.2422.

[36] D. Levesque and W. T. Ashurst. "Long-Time Behavior of the Velocity Autocorrelation Function for a Fluid of Soft Repulsive Particles". In: *Physical Reiew. Letters* 33 (5 1974), pp. 277–280. DOI: 10.1103/PhysRevLett.33.277.

[37] Qian Li. "scTour: a deep learning architecture for robust inference and accurate prediction of cellular dynamics". In: *bioRxiv* (2022). DOI: 10.1101/2022.04.17.488600.

[38] Xavier Michalet. "Mean square displacement analysis of single-particle trajectories with localization error: Brownian motion in an isotropic medium". In: *Physical Review E* 82 (2010). DOI: 10.1103/PhysRevE.82.041914.

[39] Naveen Michaud-Agrawal et al. "MDAnalysis: A toolkit for the analysis of molecular dynamics simulations". In: *Journal of Computational Chemistry* 32.10 (2011), pp. 2319–2327. DOI: https://doi.org/10.1002/jcc.21787.

[40] Mohammad Rafiq Muqri, Eric John Wilson, and Javad Shakib. "A Taste of Python – Discrete and Fast Fourier Transforms". In: *2015 ASEE Annual Conference amp Exposition*. 10.18260/p.23464. https://peer.asee.org/23464. Seattle, Washington: ASEE Conferences, 2015.

[41] Handan Olgar and Wolfhard Janke. "Gyration tensor based analysis of the shapes of polymer chains in an attractive spherical cage". In: *The Journal of chemical physics* 138 (Feb. 2013). DOI: 10.1063/1.4788616.

[42] Fernando A. Oliveira et al. "Anomalous Diffusion: A Basic Mechanism for the Evolution of Inhomogeneous Systems". In: *Frontiers in Physics* (2019). DOI: 10.3389/fphy.2019.00018.

[43] Fernando A. Oliveira et al. "Anomalous Diffusion: A Basic Mechanism for the Evolution of Inhomogeneous Systems". In: *Frontiers in Physics* 7 (2019). DOI: 10.3389/fphy.2019.00018. URL: https://www.frontiersin.org/article/10.3389/fphy.2019.00018.

[44] Riccardo Raccis et al. "Confined Diffusion in Periodic Porous Nanostructures". In: *ACS Nano* 5.6 (2011), pp. 4607–4616. DOI: 10.1021/nn200767x.

[45] Daniel Roe and Thomas Cheatham. "PTRAJ and CPPTRAJ: Software for Processing and Analysis of Molecular Dynamics Trajectory Data". In: *Journal of Chemical Theory and Computation* 9.7 (2013), pp. 3084–3095. DOI: 10.1021/ct400341p.

[46] JOSEPH RUDNICK and GEORGE GASPARI. "The Shapes of Random Walks". In: *Science (American Association for the Advancement of Science)* 237.4813 (1987), pp. 384–389.

[47] Rubén San-Segundo et al. "Parkinson's Disease Tremor Detection in the Wild Using Wearable Accelerometers". In: *Sensors* 20.20 (2020). DOI: https://doi.org/10.3390/s20205817.

[48] Trifce Sandev, Ralf Metzler, and Aleksei Chechkin. "From continuous time random walks to the generalized diffusion equation". In: *Fractional Calculus and Applied Analysis* 21.1 (2018), pp. 10–28. DOI: doi:10.1515/fca-2018-0002.

[49] M. A. F. dos Santos, Luis Menon Junior, and Danilo Cius. *Superstatistical approach of the anomalous exponent for scaled Brownian motion.* 2022. DOI: 10.48550/ARXIV.2206.07820. URL: https://arxiv.org/abs/2206.07820.

[50] Michael J. Saxton. "Anomalous Subdiffusion in Fluorescence Photobleaching Recovery: A Monte Carlo Study". In: *Biophysical Journal* 81.4 (2001), pp. 2226–2240. ISSN: 0006-3495. DOI: https://doi.org/10.1016/S0006-3495(01)75870-5.

[51] Michael J. Saxton and Ken Jacobson. "SINGLE-PARTICLE TRACKING: Applications to Membrane Dynamics." In: *Annual Review of Biophysics and Biomolecular Structure* 26.1 (1997), p. 373.

[52] Skipper Seabold and Josef Perktold. "statsmodels: Econometric and statistical modeling with python". In: *9th Python in Science Conference*. 2010.

[53] Steven H. Strogatz. *Nonlinear Dynamics and Chaos: With Applications to Physics, Biology, Chemistry, and Engineering: With Applications to Physics, Biology, Chemistry, and Engineering*. 2nd ed. Westview Press, 2014, p. 513.

[54] Karel vSolc. "Shape of a Random-Flight Chain". In: *Journal of Chemical Physics* 55 (1971), pp. 335–344.

[55] Jiří Vymětal and Jiří Vondrášek. "Gyration- and Inertia-Tensor-Based Collective Coordinates for Metadynamics. Application on the Conformational Behavior of Polyalanine Peptides and Trp-Cage Folding". In: *The Journal of Physical Chemistry A* 115.41 (2011), pp. 11455–11465. DOI: 10.1021/jp2065612.

[56] Thorsten Wagner et al. "Classification and Segmentation of Nanoparticle Diffusion Trajectories in Cellular Micro Environments". In: *PLOS ONE* 12.1 (Jan. 2017), pp. 1–20. URL: https://doi.org/10.1371/journal.pone.0170165.

[57] Stephen R. Williams et al. "Velocity Autocorrelation Functions of Hard-Sphere Fluids: Long-Time Tails upon Undercooling". In: *Physical Review Letters* 96 (8 2006). DOI: 10.1103/PhysRevLett.96.087801.

[58] Xiao-Lun Wu and Albert Libchaber. "Particle Diffusion in a Quasi-Two-Dimensional Bacterial Bath". In: *Physical Review Letters* 84 (13 2000), pp. 3017–3020. DOI: 10.1103/PhysRevLett.84.3017.

[59] Wang Xingyuan, Luo Chao, and Meng Juan. "Nonlinear dynamic research on EEG signals in HAI experiment". In: *Applied Mathematics and Computation* 207.1 (2009), pp. 63–74. DOI: https://doi.org/10.1016/j.amc.2007.10.064.

[60] V. Zaburdaev, S. Denisov, and J. Klafter. "Lévy walks". In: *Review of Modern Physics* 87 (2 2015), pp. 483–530. DOI: 10.1103/RevModPhys.87.483.
